# Simulating climate change *in situ* in a tropical rainforest understorey using active air warming and CO_2_ addition

**DOI:** 10.1002/ece3.8406

**Published:** 2022-01-24

**Authors:** Maaike Y. Bader, Elodie Moureau, Nada Nikolić, Thomas Madena, Nils Koehn, Gerhard Zotz

**Affiliations:** ^1^ Faculty of Geography Ecological Plant Geography University of Marburg Marburg Germany; ^2^ Institute for Biology and Environmental Sciences Functional Ecology of Plants University of Oldenburg Oldenburg Germany; ^3^ Faculty of Natural Sciences Electronics Workshop University of Oldenburg Oldenburg Germany

**Keywords:** climate‐change experiment, CO_2_ enrichment, epiphytes, forced‐air warming, forest understorey, open‐top chamber, tropical rainforest

## Abstract

Future climate‐change effects on plant growth are most effectively studied using microclimate‐manipulation experiments, the design of which has seen much advance in recent years. For tropical forests, however, such experiments are particularly hard to install and have hence not been widely used. We present a system of active heating and CO_2_ fertilization for use in tropical forest understoreys, where passive heating is not possible. The system was run for 2 years to study climate‐change effects on epiphytic bryophytes, but is also deemed suitable to study other understorey plants. Warm air and CO_2_ addition were applied in 1.6‐m‐tall, 1.2‐m‐diameter hexagonal open‐top chambers and the microclimate in the chambers compared to outside air. Warming was regulated with a feedback system while CO_2_ addition was fixed. The setup successfully heated the air by 2.8 K and increased CO_2_ by 250 ppm on average, with +3 K and +300 ppm as the targets. Variation was high, especially due to technical breakdowns, but not biased to times of the day or year. In the warming treatment, absolute humidity slightly increased but relative humidity dropped by between 6% and 15% (and the vapor pressure deficit increased) compared to ambient, depending on the level of warming achieved in each chamber. Compared to other heating systems, the chambers provide a realistic warming and CO_2_ treatment, but moistening the incoming air would be needed to avoid drying as a confounding factor. The method is preferable over infrared heating in the radiation‐poor forest understorey, particularly when combined with CO_2_ fertilization. It is suitable for plant‐level studies, but ecosystem‐level studies in forests may require chamber‐less approaches like infrared heating and free‐air CO_2_ enrichment. By presenting the advantages and limitations of our approach, we aim to facilitate further climate‐change experiments in tropical forests, which are urgently needed to understand the processes determining future element fluxes and biodiversity changes in these ecosystems.

## Graphical abstract

We introduce an effective experimental system for studying plant responses to warming and CO_2_ enrichment *in situ* in tropical rainforests using open‐top chambers and forced‐air warming. The system is suitable for treating shrubs, saplings, and epiphytes, while ecosystem‐level studies may need infrared heating and free‐air CO_2_ enrichment. Developing *in situ* experiments in rainforests is urgently needed to help understand the response of these ecosystems to global change.

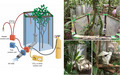

## 1 INTRODUCTION

Experiments manipulating microclimatic conditions have become important tools in ecological research, aiming at understanding the consequences of climate change for physiological processes, biodiversity, and ecosystem functioning (Aronson & McNulty, 2009; De Boeck et al., 2015; Norby et al., 2007). Microclimate manipulation generally involves increased ambient or soil temperatures, sometimes combined with CO_2_ fertilization or changes in water supply (Ettinger et al., 2019; Mikkelsen et al., 2007). Most experiments focus on agricultural systems (Ainsworth et al., 2008), but a substantial literature has also accumulated for natural vegetation, in particular for mid‐to‐high latitudes. Here, such experiments have provided a wealth of information about physiological and ecological responses of plants and, to a lesser extent, animals to different climate factors and their interactions (Pelini et al., 2011; Walker et al., 2006).

Tropical forests, in particular, have not seen a lot of climate manipulation experiments (Cavaleri et al., 2015), even though they are a biome exchanging more CO_2_ with atmosphere than any other (Beer et al., 2010; Hubau et al., 2020), so that climate‐induced modifications of these ecosystems can feed back strongly to the global climate. Moreover, tropical species may be more sensitive to changes because of limited temperature acclimation abilities (Cunningham & Read, 2003; Janzen, 1967), and for some groups (including ectothermic animals and trees), it has been shown that they exist close to or already above their physiological optimum temperature (Doughty & Goulden, 2008; Tewksbury et al., 2008). The ecological importance and potential vulnerability of tropical plants, and even more so tropical ecosystems, are not reflected in the number of climate‐change experiments. A few studies have addressed the responses of tropical forest tree leaves or branches (Doughty, 2011; Würth et al., 1998b) or understorey plants (Würth et al., 1998a) to warming and/or CO_2_ enrichment in situ. However, most climate‐change studies on rainforest species have used potted seedlings, saplings, or other plants (Cheesman & Winter, 2013; Fauset et al., 2019; Granados & Körner, 2002; Krause et al., 2013; Slot & Winter, 2017; Wagner & Zotz, 2018; Winter & Virgo, 1998), or artificial, ex situ communities (Körner & Arnone, 1992; Reekie & Bazzaz, 1989). Although certain patterns have emerged, the number of in situ experiments in particular is still much too small to lift predictions of climate‐change effects on tropical forest plants, let alone forests, out of the realm of speculation (Cavaleri et al., 2015; Körner, 2004; Lloyd & Farquhar, 2008).

To our knowledge, in tropical rainforests, only one operational system exists to date for warming and one for CO_2_ fertilization. The warming study uses infrared lamps to warm understorey plants and soil in the subtropical wet forest of Luquillo Experimental Forest on Puerto Rico (Kimball et al., 2018). The CO_2_ enrichment is achieved in open‐top chambers (OTC) and serves as a pilot study for a planned forest‐level free‐air CO_2_ enrichment (FACE) experiment in the Amazon (the AmazonFACE, Hofhansl et al., 2016). The lack of climate‐change experiments in tropical forests is not only due to the generally lower research investment in tropical regions but also to the logistic and technical challenges of installing such experiments in this environment. Logistic challenges, both for large‐scale and small‐scale experiments, include the availability and reliability of power, CO_2_, and other supplies. Technical challenges are posed by the high humidity, leading to fast corrosion and fast growth of algae and fungi, the abundance of cable‐eating fauna, the size of the trees, and the dark conditions in the understorey, precluding the use of passive warming systems (Cheesman & Winter, 2013; Kimball et al., 2018).

In principle, warming experiments may use passive or active warming. Passive warming is generally achieved in chambers, sometimes closed but more commonly OTCs, which allow the air to warm in the sun by reducing convection (Bokhorst et al., 2011). Full sky exposure and low‐stature vegetation are critical conditions to enable such solar heating. Even though such passive heating systems tend to lead to canopy cooling during the night and vary in their effectiveness with the seasons, they are still an effective and cost‐efficient method, widely used, for example, in tundra and grassland ecosystems (e.g., Bokhorst et al., 2011; De Boeck et al., 2015; Walker et al., 2006).

Active warming allows better control over the level and location of heating and can be applied in vegetation where solar input is insufficient for passive warming. Possible methods include infrared lamps, heating cables, or supplying warm air in open‐top or closed‐top chambers (Ettinger et al., 2019; Kimball et al., 2018; Norby et al., 1997; Pelini et al., 2011; Rustad et al., 2001). An important consideration when choosing a warming method is the extent to which the method mimics expected climate change appropriately for the processes of interest (Amthor et al., 2010; Aronson & McNulty, 2009; Kimball, 2011). Generally, a spatially homogenous warming of the air and a warming of surfaces (plants, soil, etc.) via convective processes is expected under climate change. The provision of radiative heat may therefore cause artefacts unrelated to future climate change, like a heating of exposed surfaces beyond air temperature (which happens naturally in sunny but not in shady habitats) and associated superficial drying (Amthor et al., 2010; Ettinger et al., 2019). Likewise, heating with underground heating cables, the most commonly used method in forest ecosystems (Rustad et al., 2001), may cause a temperature heterogeneity that would not occur under climate change either (Aronson & McNulty, 2009). Whether these artefacts are a problem depends on the question being addressed with the experiment. Providing warm air seems a more realistic way of heating a system, although the required enclosure and air movement may also cause climatic side‐effects (Norby et al., 2007; Rustad et al., 2001). Such a system requires a feedback regulation of the energy input and can require a high input of electricity, like other active heating systems, or where possible may be powered by solar heat collection (e.g., Chiba & Terao, 2014).

CO_2_ fertilization can be provided at different scales and using different levels of isolation from the free atmosphere: In leaf chambers, controlled‐environment chambers, greenhouses, whole‐tree closed chambers, open‐top chambers, or free‐air CO_2_ enrichment (FACE) experiments (Ainsworth et al., 2008; Drake et al., 1985; Körner et al., 2005). Thereby, open‐top chambers may represent the best compromise between the alteration of microclimatic conditions and the loss of CO_2_ from the target location (Drake et al., 1985). The latter criterion is especially important in situations where CO_2_ is expensive and difficult to transport to the study site. Open‐top chambers have been used to study effects of atmospheric constituents on plants since the 1970s (e.g., Heagle et al., 1973) and for CO_2_ enrichment experiments since the 1980s (e.g., Drake et al., 1985). In open environments, they are frequently used as a warming treatment because of their effect on the microclimate. However, in shady environments, this effect is much smaller, which may make them particularly suitable for CO_2_ enrichment in forest understoreys (Würth et al., 1998a).

Tropical plants show mixed responses to CO_2_ enrichment, but in the typical deep shade of forest understoreys, the CO2 response tends to be strongest, as under such conditions plant growth is carbon limited (Granados & Körner, 2002; Körner, 2004; Winter & Virgo, 1998). Although photosynthesis should be light limited rather than CO_2_ limited in this environment, the decreased photorespiration (which is stronger under warmer conditions, i.e. in tropical lowlands) at higher CO_2_ levels prevents a critical waste of captured light energy, increasing the quantum yield and thereby carbon gain at low light (Würth et al., 1998a).

To facilitate a wider application of climate‐change experiments in tropical rainforests, we here present the technical details, production guidelines, and effectiveness of a heating and CO_2_ fertilization system we developed for a study in Costa Rica. Our study targeted bryophytes (mosses and liverworts) growing as epiphytes in the understorey of a tropical lowland rainforest. We asked how warming and increased atmospheric CO_2_ might interact to affect bryophyte carbon balances. In this shaded environment, it is not possible to achieve heating using passive OTCs. Therefore, the OTCs were actively heated, combined with a CO_2_ fertilization system in part of the chambers. This system, although here used for studying understorey epiphytes, also holds promise for the study of other co‐occurring plants like tree or liana seedlings and shrubs. The warming design is based on a system used by Cheesman and Winter (2013) for tree seedlings in Panama, although, in contrast to ours, their system was set up in a clearing and not inside the forest, was used for nighttime warming only, and used constant rather than regulated heating. In the following, we present our system and the effects of the treatments on the microclimate and CO_2_ levels in the chambers, registered over the course of 15 months (12 for CO_2_) in the rainforest understorey of La Selva Biological Station, Costa Rica.

## 2 METHODS

### 2.1 Study area

The experiment was installed at the OTS (Organisation for Tropical Studies) biological station La Selva, in northwest Costa Rica (10.431° N, 84.007° W, 60 m a.s.l.), with a typical tropical wet lowland forest climate. The annual mean temperature is 26.3°C and temperatures vary between ca. 20 and 30°C all year (Jiménez‐Rodríguez et al., 2020). The mean annual precipitation is 4350 mm (mean of 54 years), with a bimodal rainfall seasonality. July is usually the wettest month with ca. 550 mm and March the driest with still nearly 200 mm (Jiménez‐Rodríguez et al., 2020) (Figure A6).

This research station provided two important prerequisites for the experiment: power supply inside the forest, a protected forest with access only for authorized personnel, and local accommodation and laboratory facilities, necessary for the daily supervision of the experiment.

### 2.2 Experimental design

The experimental design consisted of a full‐factorial combination of warming and CO_2_ fertilization, with two control treatments: chambers supplied with ambient air and unmanipulated ambient plots. We used five replications. The targeted CO_2_ increase was 300 ppm, only during the day, and the targeted warming was 3 K, continuously day and night. These magnitudes correspond to the projected increase in global average temperature over land by 2100, relative to 1986–2005, according to the intermediate IPCC modeling scenario RCP6.0, or the upper 75% percentile of the RCP4.5 scenario for Costa Rica and much of the tropics (IPCC, 2013).

### 2.3 Experimental setup

**FIGURE 1:**
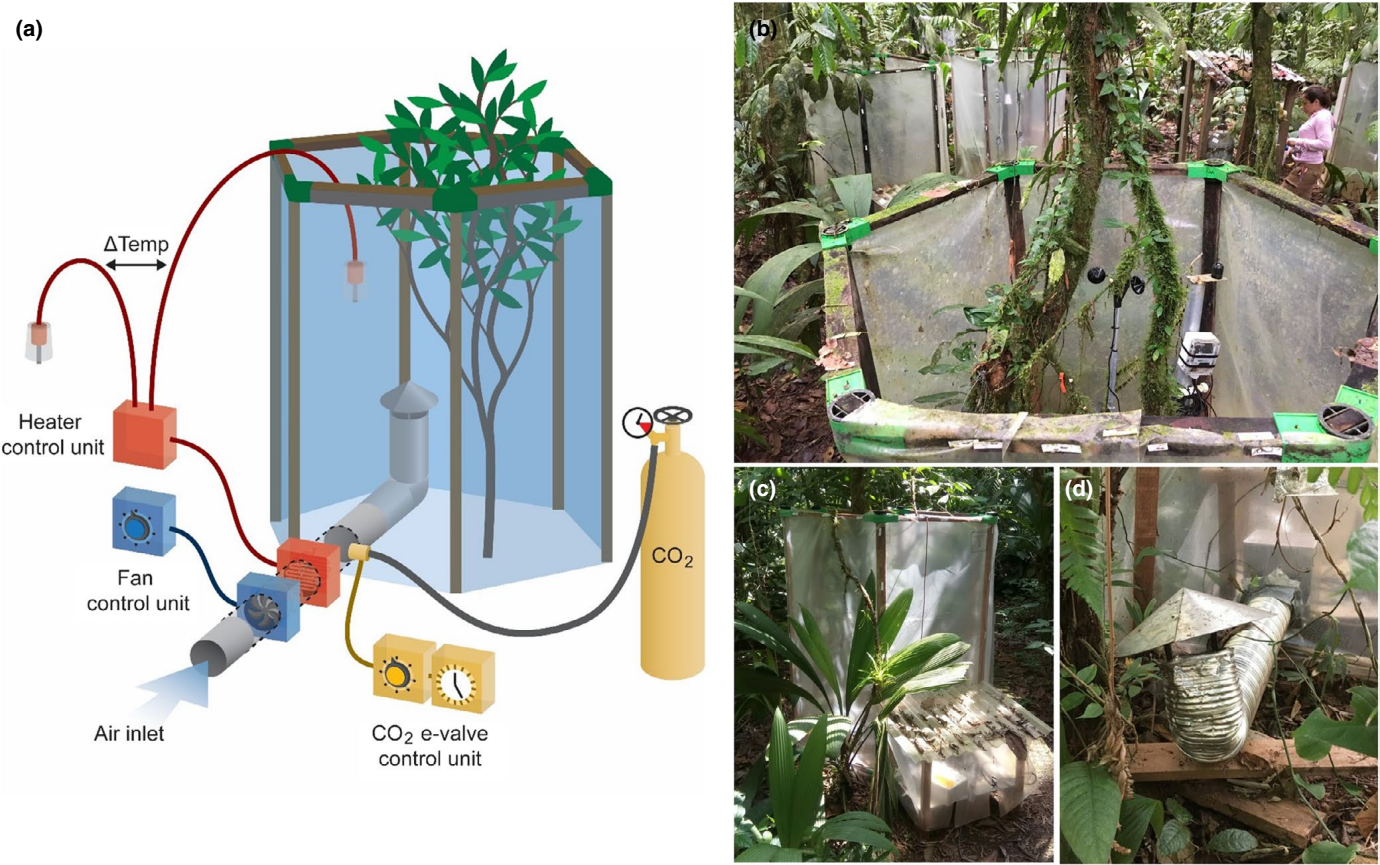
(a) Schematic overview of the installation showing an open‐top chamber around a sapling with a system providing both heating and CO_2_ enrichment. Fan speed is set in its control unit (both in blue). Incoming air is warmed by a heater (in red), regulated by its control unit via a feedback system based on the inside–outside temperature difference. An electronic valve (e‐valve, in yellow) controls the rate at which CO_2_ is added, as set in its control unit, which also includes a timer that switches off the CO_2_ supply during the night. Drawing by Marc Maas. (b) Open‐top chambers installed in the rainforest understorey at La Selva biological station, Costa Rica. Inside the chamber in the foreground, a climate station is registering wind, PAR, and leaf wetness. In the background, apart from three further chambers, a roof with the CO_2_ bottle can also be seen in front of the researcher. (c) Open‐top chamber and roofed electronics. (d) View from the inside of a chamber showing the air outlet with chimney roof and in the background, outside of the chamber, the electronics area. For details about the electronics, see Figures A2, A3, A4, A5

**FIGURE A1:**
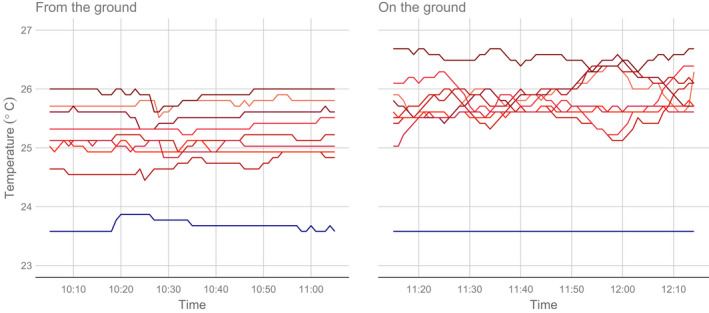
Results of one of the last tests of the heating system in the laboratory in Oldenburg. In the test we used chambers of the same size and shape as those used later in the rainforest. We tested how putting the chamber ca. 10 cm up from the ground on little feet (a) or directly on the ground (b) affected the heating effectiveness and the spatial variability in the temperature reached within the chamber. The blue line shows the outside temperature, the red lines the temperatures at different positions inside the chamber, with sensors distributed both horizontally and vertically. We did not store the exact position of each sensor but rather evaluated the general means and variability of the temperatures reached. The mean was clearly higher and closer to the intended +3 K, and the variability somewhat lower, when placing the chamber directly on the ground. This position was therefore chosen also for the field experiment

The chambers were hexagonal constructions with open tops and bottoms, set‐up directly on the forest floor (Figure 1). This position allowed for a more homogenous and more effective warming than placing the chambers at some distance off the ground (test results in Figure A1), which otherwise would be a possible configuration for studying epiphytes or other aboveground subjects. The chambers were 1.6‐m‐tall and ca. 1.2‐m‐wide vertex to vertex (0.6‐m side lengths), with ribs made of local hardwood and adjustable corners of hard plastic (Vario‐Quick, GeKaHo, Lahr‐Schwarzwald, Germany), and the sides spun with transparent plastic greenhouse foil. Keeping an open top allowed a better exchange of gases and energy with the atmosphere and an unmodified exposure to precipitation. A closed‐top chamber would enable a stronger control on the treatment but would also move the experiment away further from natural conditions. Because of the low‐incoming radiation, the chambers were expected to have relatively small effects on temperature conditions. The 20 chambers were placed in five blocks within an area of ca. 500 m^2^ in fully grown closed forest near the La Selva research station (Figure 1b). As the study was focused on epiphytes, each chamber included at least one shrub or small tree with epiphytic bryophytes on it.

### 2.4 Equipment design

**FIGURE A2:**
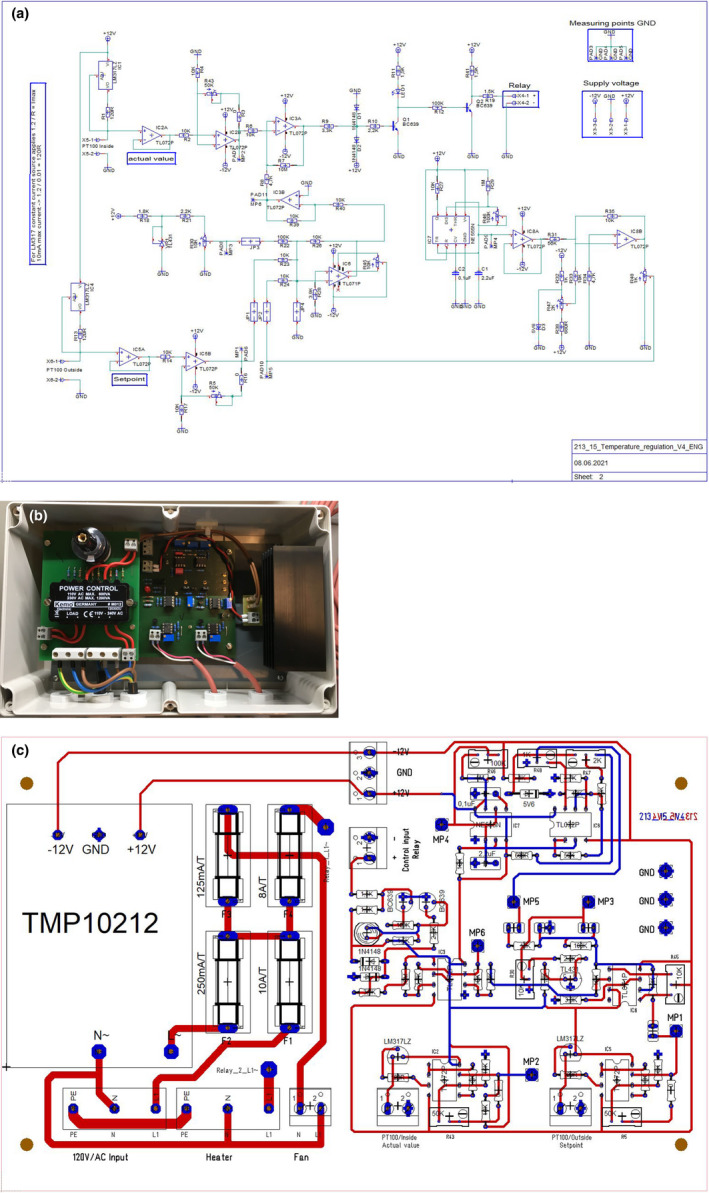
(a) Circuit diagram for the temperature control unit used to regulate the heat going into our open‐top chambers in the rainforest understorey at La Selva, Costa Rica. The temperature is regulated by switching on and off the heater coil in response to the measured temperature difference between chamber and outside air. The control unit is run with +12V/DC and −12V/DC from the TMP10212 voltage converter (see Figure A3). Two temperature sensitive resistors (PT100) with a <0.5 K precision (PT100 / KS/E 10/5, FuehlerSysteme eNET International GmbH, Nürnberg, Germany) are connected and should be installed to measure air temperature inside and outside of the chamber. The outside one (PT100‐Outside) forms the basis for the desired value (the setpoint), the inside one (PT100‐Inside) represents the actual value. The inside PT100 forms a tension divider with a second resistor (R1) to measure the chamber temperature. The tension divider voltage is amplified twice with operational amplifiers (TL072). The external temperature is measured in the same way and a voltage offset equivalent to a 3 K temperature difference (in our case; this value is set by an adjustment of the potentiometer, yellow boxes in Figure A2b,c) is added to it. The switch regulating the heating then compares these voltages, and whenever the external temperature + offset is higher than the chamber temperature the relay is closed, turning on the chamber heating. The fan for the heater was controlled seperately (see Figure A4). (b) Photo of the temperature control unit (right) and the fan control unit (left). For the temperature control unit including the power supply (below the fan regulation unit) see Figure A3b. The yellow box indicates the potentiometer with screw used to adjust the setpoint for the inside temperature. (c) Circuit board design for the temperature control unit, including the power supply for this unit and the fan and heater (see the circuit diagram in Figure A3a). The yellow box indicates the potentiometer used to adjust the setpoint for the inside temperature. Gerber files for the circuits are provided as Supporting Information

**FIGURE A3:**
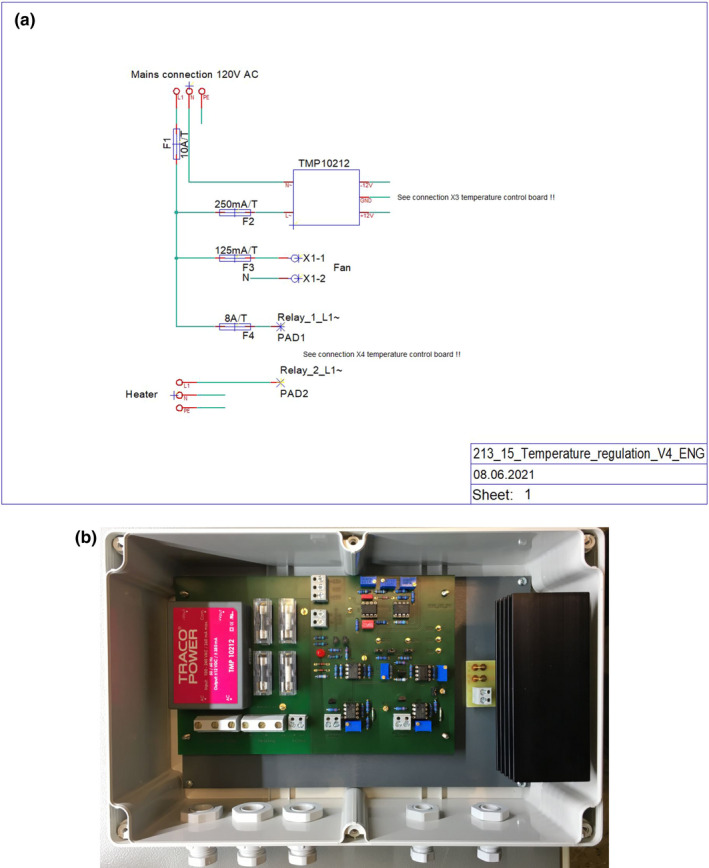
(a) Circuit diagram for the power supply of the temperature control unit and the heater and fan in our warming experiment in open‐top chambers in the rainforest understorey at La Selva, Costa Rica. The TMP10212 converter turns the 120 V/AC mains voltage (Costa Rica) into a +12 V/DC and −12 V/DC voltage, which are used to supply the temperature control unit (see Figure A2). The 120V mains voltage is also transferred via the connection terminals to the fan and the heater (see Figures A2 and A4). All connections are safeguarded with fuses, which burn at high currents and thus protect the elements of the setup against damage. (b) Photo of the power supply (left) and the temperature control unit (right)

**FIGURE A4:**
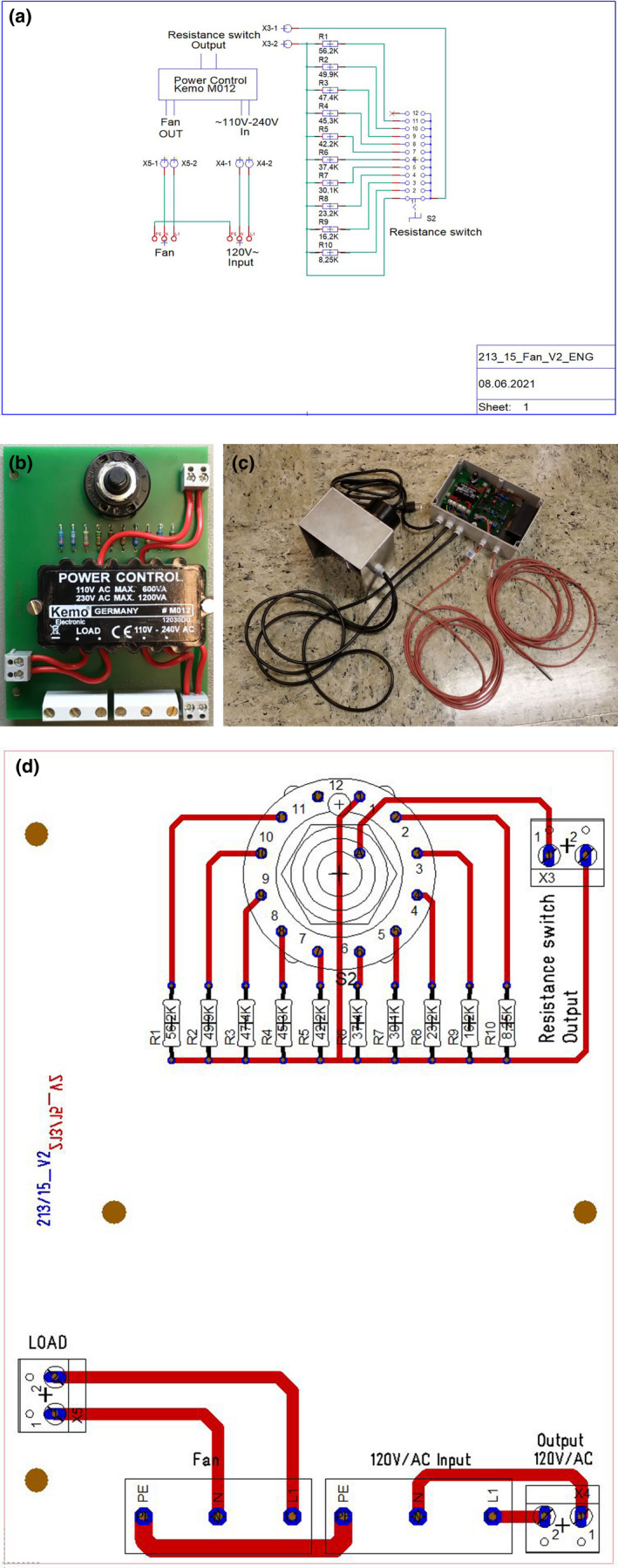
(a) Circuit diagram for the control unit for the fan blowing warmed, CO_2_ enriched or ambient air into our open‐top chambers in the rainforest understorey at La Selva, Costa Rica. Fan speed is set to a fixed value by the user. On the left a Power Control Kemo regulator (M012) is shown. This Kemo regulator runs on main voltage (120 V/AC in Costa Rica). Attached to the Kemo regulator is the connection for the fan (Fan OUT) and a resistor switch. This switch is shown on the right. It consists of a rotary switch with 12 positions, each of which is connected to a resistor with a different value (resistance). The rotary switch is then used to connect the different resistors to the Kemo regulator, so that the fan turns faster or slower (between 580 and 1380 revolutions per minute). The fan used was part of a heater (HVI 030, Stego). The heating was controlled seperately (see Figure A2), the power supply for the fan and heater is shown in Figure A3. (b) Photo of the fan regulation unit. The rotary switch is shown on the top. (c) Photo of the complete heater +fan control unit, including the heater (left, in protective box) and temperature sensors (with orange cables). (d) Circuit board design for the fan regulation unit. Gerber files for the circuits are provided as Supporting Information

**FIGURE A5:**
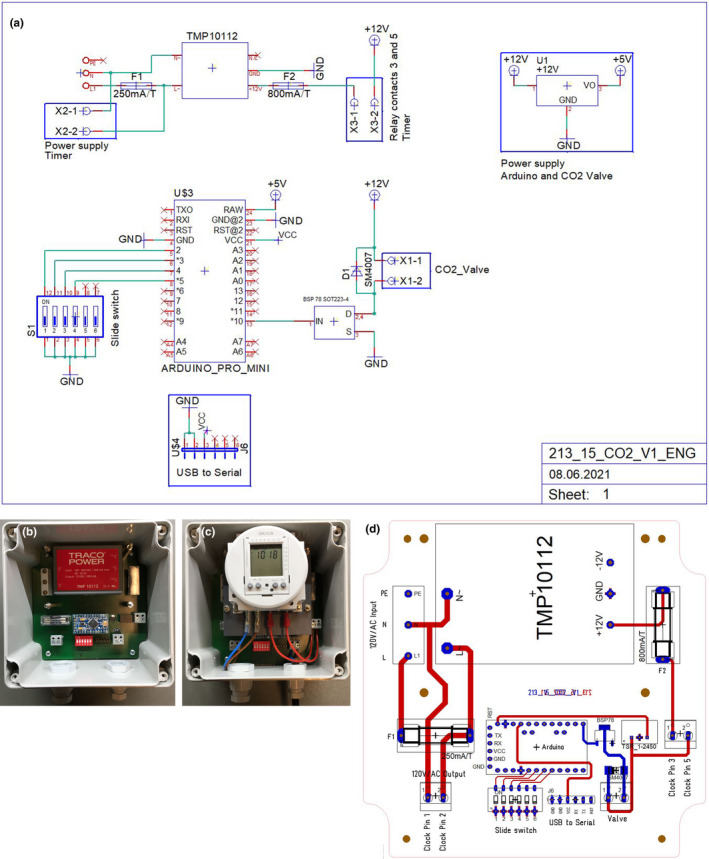
(a) Circuit diagram for the CO_2_ control unit in our CO_2_ enrichment (and warming) experiment in open‐top chambers in the rainforest understorey at La Selva, Costa Rica. CO_2_‐valve pulse intervals are set to a fixed value by the user. The TMP10212 converter converts the 120 V/AC mains voltage (Costa Rica) into a 12 V/DC voltage, which is used to supply the CO_2_ valve and a second voltage converter (TSR1‐2450) which turns 12 V/DC into 5V/DC. This 5V is used to run the microcontroller (Arduino Mini Pro). The DIP slider switch (S1 in diagram) different on / off intervals can be selected from the options stored in the microcontroller (i.e. a rough idea of the needed intervals is required to pre‐set these options, which can then be fine‐tuned in the field by monitoring the resulting CO_2_ concentrations in the open‐top chambers). The BSP78 MOS transistor on the microcontroller serves as a switch that opens or closes the electronic valve (Festo MFH‐3‐M5) according to the selected interval length, thus controlling, together with the pressure set by the regulating CO_2_ valve on the bottle, the amount of CO_2_ let into the air stream. The timer (Grässlin FMD 120) is included in the circuit to switch off the CO_2_ supply during the night, when no biological effect of interest is expected, to reduce the amount of CO_2_ used for the experiment. (b) Photo of the CO_2_ control unit inside the waterproof case. (c) Photo of the CO_2_ control unit with the timer installed, both inside the waterproof case. (d) Circuit board design for the CO_2_ control unit, including the power supply for this unit and the CO_2_ valve. Gerber files for the circuits are provided as Supporting Information

**FIGURE A6:**
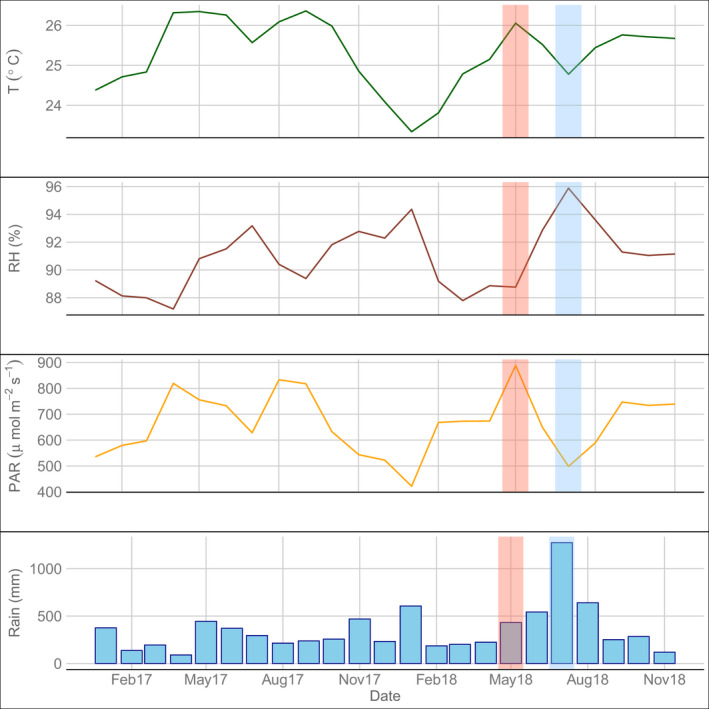
Meteorological conditions (monthly means for T, RH, and daytime PAR, monthly totals for rain) at a standard weather station in the station clearing at La Selva biological station during our investigation period. The red and blue vertical bands indicate the dry‐season and wet‐season month, respectively, presented in Figures 3 and 4 in the paper

All electronic regulators and controls were designed, built, and tested at the electronics workshop of the University of Oldenburg (Germany Figure A1). Temperature was increased by leading heated air into the chamber, emerging upwards from a 15‐cm‐diameter aluminum tube, covered by a roof, in the middle of the chamber at ca. 60 cm from the ground (Figure 1d). The air was heated by an inline coil heater (HVI 030, Stego, Schwäbisch Hall, Germany). The temperature was regulated by a feedback system based on the temperature difference between the air inside and outside of the chamber, measured with two Pt100 temperature sensors with a 0.5K accuracy (details of the electronics in Figures A2 and A3). The temperature difference was calibrated for each chamber individually after installation in the forest. All chambers, including the controls, had an air flow going into the chambers, with an air inlet outside nearby each chamber, either with or without heating and with or without added CO_2_. The air flow was provided by the heater fans, with heaters turned off in case of the non‐heated chambers (details of the regulation of the fans in Figure A4). CO_2_ was added into the air flow via an electronic valve (MFH‐3‐M5, Festo, Esslingen, Germany), which was set to the optimal pulse length and interval between pulses based on manual CO_2_ measurements before the start of the experiment (details of the CO_2_ regulation in Figure A5). These settings were the same among all heated CO_2_ chambers and among all non‐heated CO_2_ chambers, but the heated CO_2_ chambers had a longer pulses relative to the intervals to compensate for the faster loss of CO_2_ from these chambers. The correctness of the settings was checked weekly by measuring CO_2_ inside and outside of the chambers using a hand‐held CO_2_ probe (GMP343, Vaisala, Helsinki, Finland) installed for 3–5 h (with measurements recorded at 1‐min intervals) with no persons near, to avoid influencing the reading through breathing. CO_2_ was supplied from 50‐lb gas bottles (23 kg CO_2_ when full), with five bottles in use simultaneously for the 10 CO_2_‐enriched chambers. These bottles had to be exchanged approximately every 7–10 days, thus posing quite high operational costs. The walls of the chambers became covered in algae relatively fast and were cleaned every 6 months to reduce the difference in light conditions.

### 2.5 Microclimatic measurements

We recorded air temperature and relative humidity (RH) in paired measurements inside the heated chambers (*n* = 10) and at paired outside reference points for the duration of the experiment (456 days, May 2017 to Aug 2018) using standalone dataloggers with a resolution of 0.01 K and 0.01% RH and an accuracy of <0.05 K and 2% RH (up to 90% RH, accuracy linearly decreasing to a 4% at 100% RH; DK320 and DK325 HumiLog “Rugged” Dataloggers, Driesen + Kern, Bad Bramstedt, Germany). The outside reference points started out with *n* = 10 but were reduced to 8 or 9 as loggers failed temporarily or permanently. When less than 10 loggers were measuring outside conditions, the chamber data were paired (to calculate temperature increases and humidity changes) with data from the nearest reference point. We also recorded temperature and RH in three controls and two CO_2_ chambers from February to August 2018, to control for chamber effects. As more loggers were thus needed in the chambers, outside reference points, which showed very similar temperature patterns among them, were then reduced to four, and further reduced to a minimum of two after more loggers broke down. Data from each chamber were now paired with data from the nearest of these few outside measurement points. Additionally, five chambers and five ambient plots without chambers were equipped with sensors for leaf wetness (Leaf Wetness Smart Sensors, S‐LWA‐M003, Onset, Bourne, MA, USA), wind (Wind Speed Smart Sensor, S‐WSB‐M003, Onset), photosynthetically active radiation (PAR Smart Sensor, S‐LIA‐M003, Onset), global radiation (Silicon Pyranometer Smart Sensor, S‐LIB‐M003, Onset), and rain (in plots only, David 0.2 mm Smart Sensor, S‐RGD‐M002, Onset), registering microclimate every 15 min by Hobo Micro Stations (H21‐002 4‐Channel Data Logger, Onset).

For analyzing chamber and treatment effects on the microclimate, we used the sensors as replicates and tested for differences in means (for the whole period or for each hour of the day) using paired *t*‐tests for paired inside–outside measurements or unpaired *t*‐tests for differences between chambers. For comparing PAR levels, where only three pairs of sensors produced reliable data, we additionally tested the differences between paired inside and outside sensors with a paired *t*‐test based on hourly means on all days with measurements (*n* = 187 or 244) to avoid missing effects due to the low replication of sensors. We here report only the microclimatic effects of the setup, while the biological, bryophyte‐specific results will be reported elsewhere. We use °C for temperature and K for temperature differences. All plotting and analyses were done in R, version 4.02 (R Core Team, 2020).

## 3 RESULTS

### 3.1 Chamber effects

**FIGURE 2:**
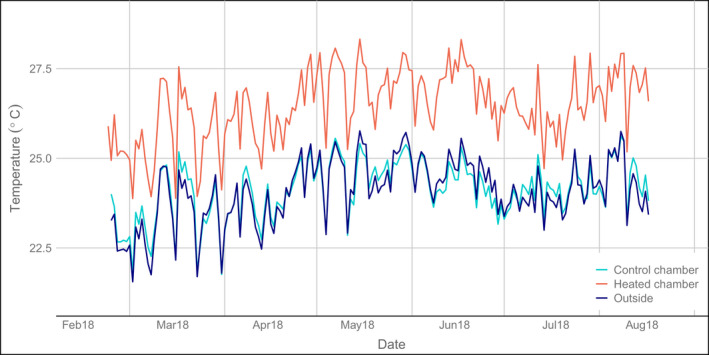
Daily mean temperatures of five heated open‐top chambers (red line, set temperature increase: 3 K), five control chambers (turquoise line), and two outside control measurements (dark blue line) in a tropical rainforest understorey at La Selva, Costa Rica, from 22‐02‐2018 to 17‐08‐2018 (data from the control chambers are available only for this period and for a longer time series of a heated chamber see Figure A10)

**FIGURE 3:**
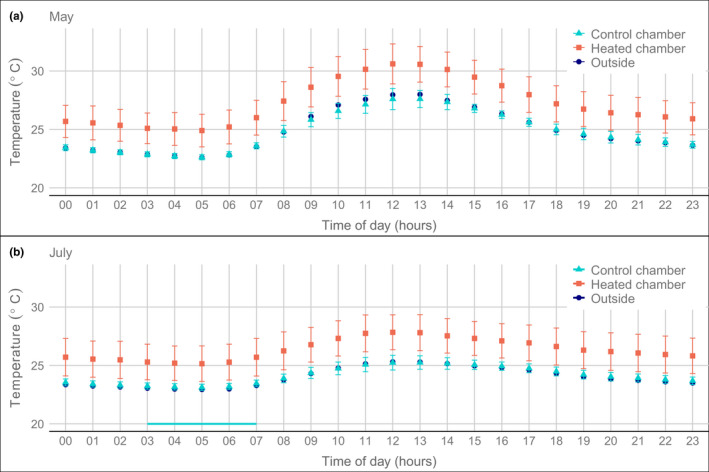
Diel pattern of temperature (hourly means) inside and outside of open‐top chambers in the rain forest of La Selva, Costa Rica. Chambers were either subjected to warming (heated) or just had a light flow of ambient air (control). Data from (a) May 2018 (31 days, dry season, see Figure A6) and (b) July 2018 (31 days, wet season). Presented are average values over all days in the respective months for ten heated chambers, five unheated chambers, and two outside measurements (see Table 1). Error bars show the standard deviation between chambers calculated per day and shown as the mean of all days, while no error bars are shown for the outside measurements due to the low replication of sensors. The turquoise horizontal line indicates a period of the day with significant differences between control chambers and outside air (warmer inside, paired *t*‐test, *p* < .05, see Table A1). The difference of the heated chambers with the control chambers and the outside air was significant all day

The control chambers did not differ in mean temperature from the outside air, with, on average, 24.1°C (± 0.2°C between plots) outside and 24.2°C (± 0.4°C between chambers) inside (Table 1). The variation in daily mean temperatures inside the control chambers and outside followed a similar pattern during the whole study (Figure 2). The diel patterns of hourly mean temperature were also very similar between outside air and control chambers, both in the dry and wet season (Figure 3). In the tested dry season month (Figure 3a), temperatures did not differ significantly at any time of the day (Table A1a), while in the wet season month (Figure 3b) temperatures were higher by 0.2K inside the control chambers than outside some early morning hours (*p* < .05, Table A1b), with no significant difference for the rest of the day.

**TABLE 1:** Mean temperature (Temp) and relative humidity (RH) in open‐top chambers in the rain forest of La Selva, Costa Rica, subjected to warming (T°C), CO_2_ enrichment (CO_2_), both or just a light flow of ambient air (control). Data were recorded every 15 min for 178 days, from 03‐02‐2018 to 17‐08‐2018, for the non‐heated chambers (CO_2_ and control), and for 456 days, from 18‐05‐2017 to 17‐08‐2018, for the heated chambers (T°C and T°C + CO_2_). Also shown are the mean differences with the outside air (ΔTemp and ΔRH)

Chamber	Treatment	Mean Temp (°C)	Mean ± *SD* ΔTemp (K)	Mean daily Min–Max Temp (°C)	Mean RH (%)	Mean daily min RH (%)	Mean ± SD ΔRH (%)
C1	CO_2_	24.1	−0.1 ± 0.8	22.2–26.6	98.3	94.4	−0.3 ± 3.2
C2	CO_2_	24.1	0.0 ± 0.5	22.3–26.6	99.3	96.1	0.7 ± 1.6
Con‐1	Control	24.1	0.1 ± 0.5	22.3–26.6	99.3	96.0	0.4 ± 1.2
Con‐2	Control	24.2	−0.1 ± 0.5	22.4–26.9	99.5	96.5	0.6 ± 2.0
Con‐3	Control	24.4	0.3 ± 0.6	22.4–27.6	98.5	93.3	0.1 ± 1.9
T‐1	T°C	27.9	3.8 ± 1.6	24.5–31.8	83.7	72.9	−15.2 ± 6.8
T‐2	T°C	25.9	1.8 ± 1.3	23.4–28.7	91.9	85.1	−6.8 ± 6.1
T‐3	T°C	26.5	2.4 ± 1.3	23.8–30.2	90.0	80.4	−8.6 ± 5.1
T‐4	T°C	27.4	3.3 ± 1.5	24.4–30.9	86.1	77.1	−12.6 ± 7.0
T‐5	T°C	25.9	1.7 ± 0.9	23.5–29.1	93.2	85.5	−5.8 ± 4.1
TC‐1	T°C + CO_2_	27.3	3.2 ± 1.4	24.7–30.7	87.0	78.3	−12.0 ± 6.2
TC‐2	T°C + CO_2_	26.0	1.9 ± 1.1	23.6–29.2	91.7	83.7	−7.2 ± 4.7
TC‐3	T°C + CO_2_	26.2	2.0 ± 1.1	23.7–29.4	91.5	82.7	−7.7± 5.2
TC‐4	T°C + CO_2_	25.9	1.8 ± 1.6	23.4–29.1	92.6	84.9	−6.5 ± 6.4
TC‐5	T°C + CO_2_	26.7	2.4 ± 1.7	24.2–30.0	88.6	80.3	−10.1 ± 7.6

**FIGURE 4:**
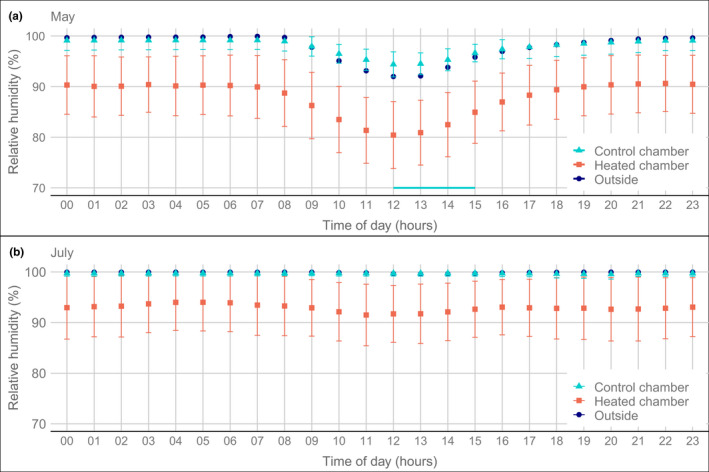
Diel pattern of relative humidity (hourly means) inside and outside of open‐top chambers in the rain forest of La Selva, Costa Rica. Chambers were either subjected to warming (heated) or just had a light flow of ambient air (control). Data from (a) May 2018 (31 days, dry season) and (b) July 2018 (31 days, wet season). Presented are average values over all days in the respective months for 10 heated chambers, five unheated chambers, and two outside measurements (see Table 1). Error bars show the standard deviation between chambers, calculated per day, and shown as the mean of all days, while no error bars are shown for the outside measurements due to the low replication of sensors. The turquoise horizontal line indicates a period of the day with significant differences between control chambers and outside air (higher RH inside, paired *t*‐test, *p* < .05, see Tables A2a,b). The difference of the heated chambers with the control chambers and the outside air was significant all day

**FIGURE A7:**
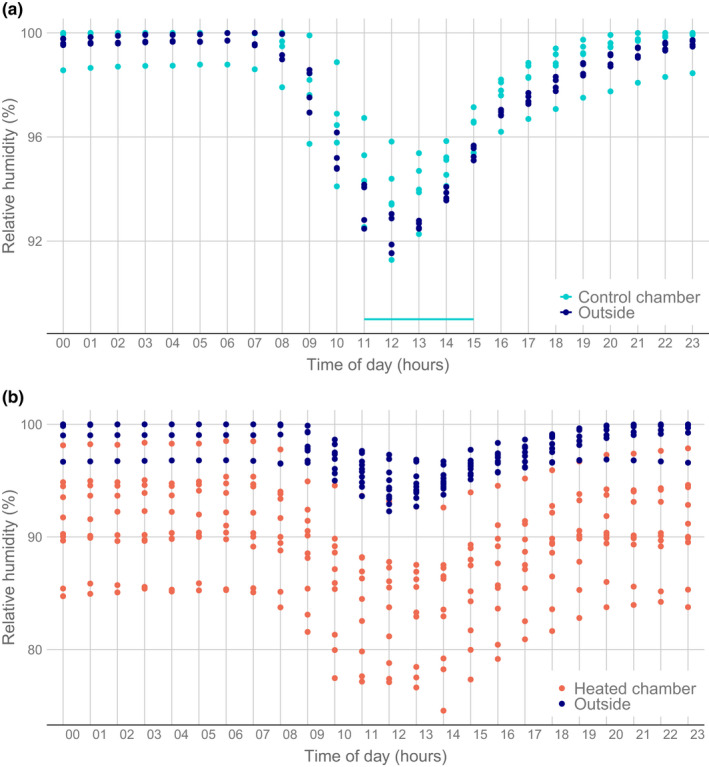
(a) Diel pattern of relative humidity (hourly means) of open‐top chambers and paired outside reference points in the rain forest of La Selva, Costa Rica, of (a) the five control chambers in April 2018 (means of 30 days), and (b) the 10 heated chambers in February 2018 (28 days). In (a), outside data are based on two measurement points used in five pairs, so outside data are replicated. Means based on the same sensors can differ when some measurements were left out when the paired chamber measurement were incomplete. The RH was significantly higher in the chambers than outside around noon (turquoise line, 11 a.m. to 2 p.m., paired *t*‐test, *p* < .05, Table A2c). In (b), we show the last month with nine outside loggers, installed until Feb 22nd, with four loggers after that, as sensors were moved to the control chambers. The difference between the heated chambers and the outside air was significant all day

**FIGURE A8:**
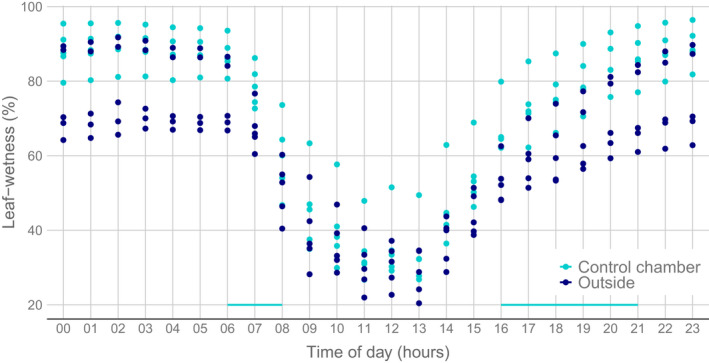
Diel patterns of leaf wetness (in %, hourly means) of five control open‐top chambers and five outside plots in the rain forest of La Selva, Costa Rica, in May 2017 (15 days – after this period the readings became unreliable due to algal growth on the artificial leaves). The turquoise line indicates where leaf wetness was significantly higher (paired *t*‐test, *p* < .05, Table A3) in the chambers than outside

Likewise, the hourly averages of relative humidity (RH) and leaf wetness were very similar between outside air and control chambers, with some differences only in the drier season (Figure 4, Figures A7 and A8). Both in April (Figure A3a) and May (Figure 4a), the chambers had a significantly higher RH than the outside air around midday (11–15 h and 12–15 h, respectively, in the 2 months) by about 2%, although there was overlap (Figure 4a, Figure A3a, Tables A2a,c). In the very wet month of July 2018, RH stayed very close to 100% both inside and outside the control chambers (Figure 4b). Perhaps as a consequence, leaf wetness (evaluated only for May 2017, before sensors were overgrown with algae and readings became unreliable) was significantly higher (*p* < .05) in the chambers than outside in the late afternoon and early evening (4–9 p.m.), and also in the early morning (6–7 a.m.), by about 10%–15% (Figure A8, Table A3). Comparing them to the effects in the heated chambers (see below), we consider that these chamber effects were negligible.

**TABLE 2:** Average CO_2_ concentration inside and outside of CO_2_‐fertilised open‐top chambers in the rain forest of La Selva, Costa Rica, additionally subjected to warming (T°C + CO_2_) or not (CO_2_). Data were recorded weekly for 12 months from Sept 2017 to Aug 2018, total *n* = 89 days, each with 3–5 h of every minute measurements. Also shown are the means and standard deviations of the differences between the chambers and the paired measurements in outside air (Mean ± *SD* ΔCO_2_)

Chamber	Treatment	Mean [CO_2_] (ppm) inside [outside]	Mean ± *SD* ΔCO_2_ (ppm)
C1	CO_2_	785 [460]	+325 ± 256
C2	CO_2_	731 [441]	+289 ± 223
C3	CO_2_	745 [463]	+282 ± 179
C4	CO_2_	865 [463]	+402 ± 277
C5	CO_2_	671 [443]	+227 ± 206
TC‐1	T°C + CO_2_	659 [488]	+172 ± 152
TC‐2	T°C + CO_2_	673 [465]	+208 ± 170
TC‐3	T°C + CO_2_	579 [447]	+131 ± 126
TC‐4	T°C + CO_2_	753 [465]	+288 ± 211
TC‐5	T°C + CO_2_	626 [460]	+166 ± 121

Wind speeds were always very low, mostly 0, both inside and outside of the chambers. Rain, or rather, throughfall amounts were spatially very variable with no reason to expect a bias related to the chambers. As the plastic foil used had a light transmission <100% and was covered to various degrees in algae through time, we expected a corresponding decrease in diffuse radiation. However, this difference was indistinguishable from the high background variability in light conditions in the forest understorey: PAR did not differ significantly between inside control chambers and outside overall (Table A4a), while for individual pairs of measurements, the differences found had no consistent direction: sometimes inside values were higher and sometimes outside values (Tables A2a, A2b, A2c).

### 3.2 Heating effectiveness

**FIGURE 5:**
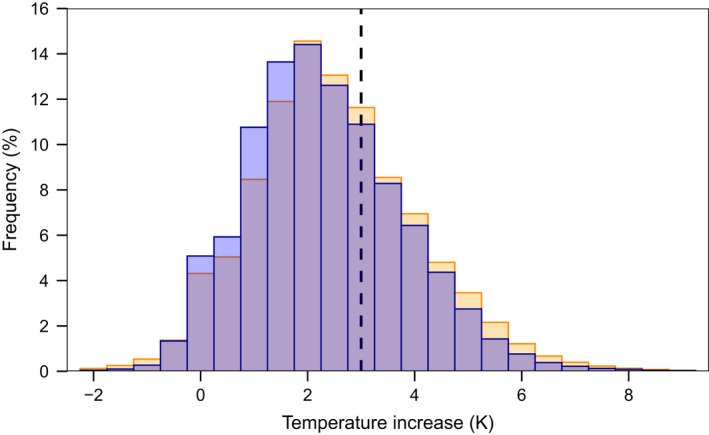
Frequency of the measured temperature increase achieved in 10 open‐top warming chambers (set temperature increase: 3 K) during the day (6 a.m. to 6 p.m.), in yellow, and night, in blue. Purple bars indicate an overlap. Frequencies were calculated based on measurements taken every 15 min for 456 days (18‐05‐2017 to 17‐08‐2018)

**FIGURE A9:**
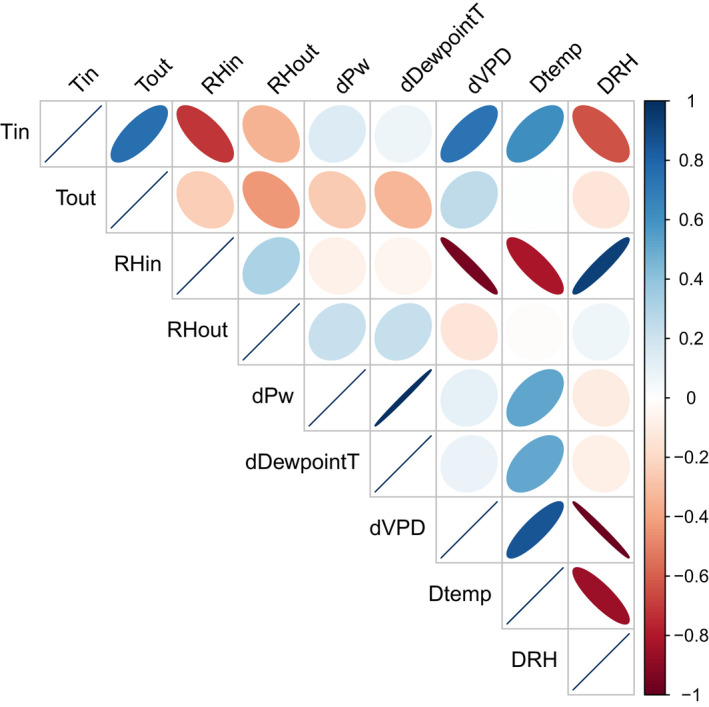
Correlation matrix for the temperature and humidity conditions inside and outside the heated chambers. The darker and narrower blue and red ellipses indicate stronger positive and negative Spearman correlations between the variables. Tin = temperature (T) inside the heated chambers, Tout = ambient T, RHin = relative humidity (RH) inside the heated chambers, RHout = ambient RH, dPw = difference in vapour pressure (absolute humidity), dDewpointT = difference in dewpoint temperature, dVPD = difference in vapour pressure deficit, Dtemp = difference in temperature, DRH = difference in RH – all differences are between the heated chambers and ambient conditions. Correlations were calculated based on measurements taken every 15 min for 456 days (18‐05‐2017 to 17‐08‐2018) in 10 chambers (heating and heating + CO_2_ addition) and the outside reference points nearest to each chamber

Temperature increased significantly in all heated chambers, on average by 1.7 to 3.8 K, depending on the chamber (paired *t*‐test, *t* = 10.2, *df* = 9, *p* < .001, Table 1). The temperature increase was within 0.5 K from the set +3 K 22% of the time. The heating achieved was less than 1 K 16% (day) to 18% (night) of the time, and more than 4 K 13% (night) to 16% (day) of the time. The heating efficiency was similar during the night and day, although overheating was more frequent during the day and underheating during the night (Figure 5). The temperature increase was unrelated to the outside temperature or RH (Spearman correlations with rho = .001 and .018, respectively, Figure A9).

**FIGURE A10:**
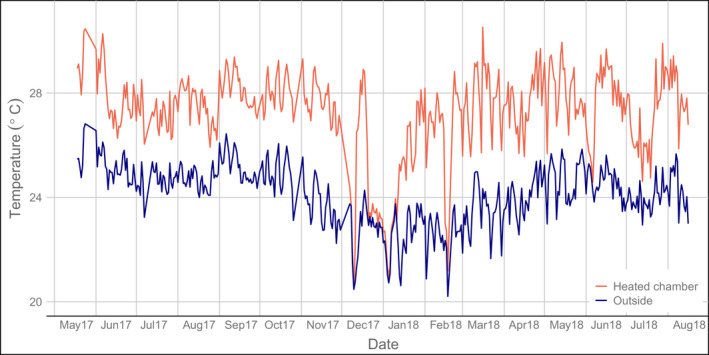
Daily mean air temperatures of one of the heated open‐top chambers (chamber TC‐1) and a paired measurement of outside air in the understorey of the rainforest at La Selva biological station, Costa Rica, during the entire study period from 18‐05‐2017 to 17‐08‐2018. The set value for the level of warming was 3 K. The disappearance of the temperature difference on several occasions was due to technical failure (e.g. burned fuses) of the equipment

**FIGURE A11:**
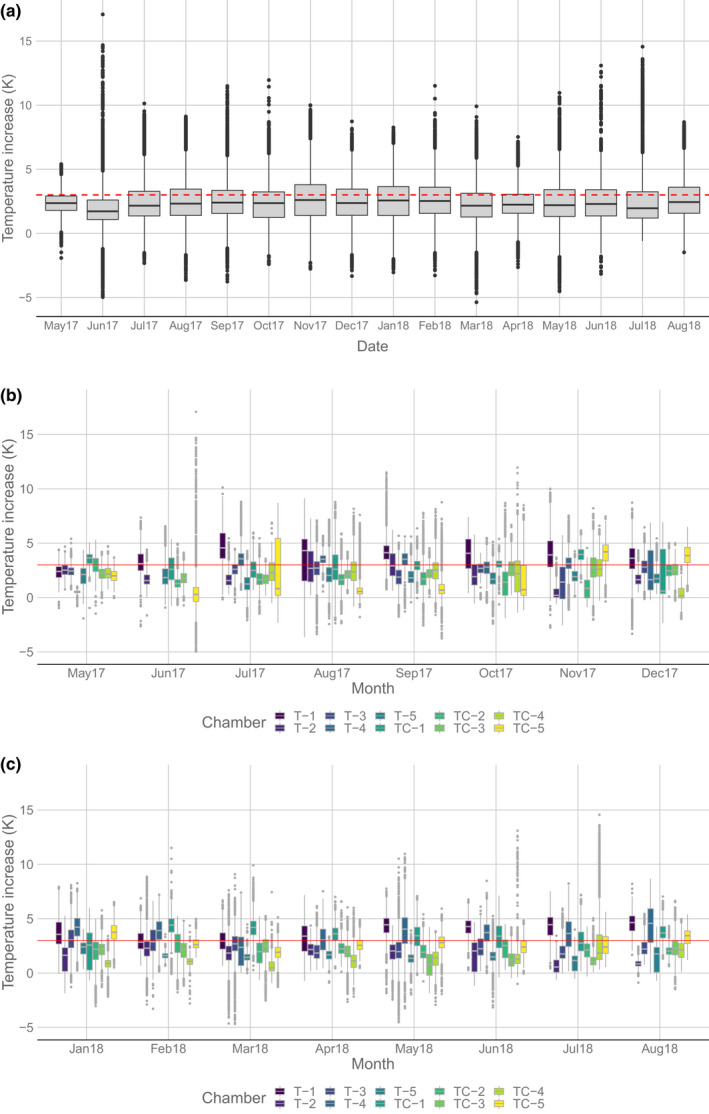
Monthly temperature increase in 10 heated open‐top chambers compared to outside air (i.e. ambient conditions) in a tropical rainforest understorey at La Selva, Costa Rica. Shown are the variation in measured values (recordings every 15‐min) per month for all chambers taken together (a), and per chamber, in the first (2017, b) and second (2018, c) half of the experiment (data split to improve visibility). Most of the variation between months is caused by technical failures, reducing the heating in one or more chambers. Variation between chambers within months is due to a combination of differences in the calibration of the feedback regulation system as well as the mentioned technical failures. The red lines indicate the targeted temperature difference (+3 K). (b and c) Boxplot of ΔTemp per chamber (colours) and month for 2017 (b) and 2018 (c). See caption above

The temperature inside the chambers followed the outside temperature closely (Figure 2, Figure A10), although in individual chambers there were temporary deviations, largely due to technical failures (mostly burned fuses, protecting the system from getting damaged due to spikes in the power supply) (Figure A10). This probably explains the rather large variation in the mean temperature difference between chambers, especially in some breakdown‐prone months (Figure A11). A second reason may be differences in the calibration of the control circuits, leading to consistent chamber‐specific temperature increases (Figure A11b,c). Additionally, the relatively low accuracy of the temperature sensors in the control system (0.5 K) could also have introduced additional variation, which could be easily avoided in future setups by using more accurate sensors. Still, in spite of such technical problems, a clear temperature increase was achieved for most of the time (Figure 5) and on average the temperature increase was close to the intended 3 K. During the daytime, higher temperature differences, also beyond the intended 3 K, were observed more frequently than during the night. This may be due to additional heating by direct sunlight, which, even in the shady understory, could reach some chambers through canopy gaps for up to a few hours a day. The spatial variability within the chamber amounted to about 1 K in the test setup (Figure A1).

### 3.3 Air humidity

**FIGURE 6:**
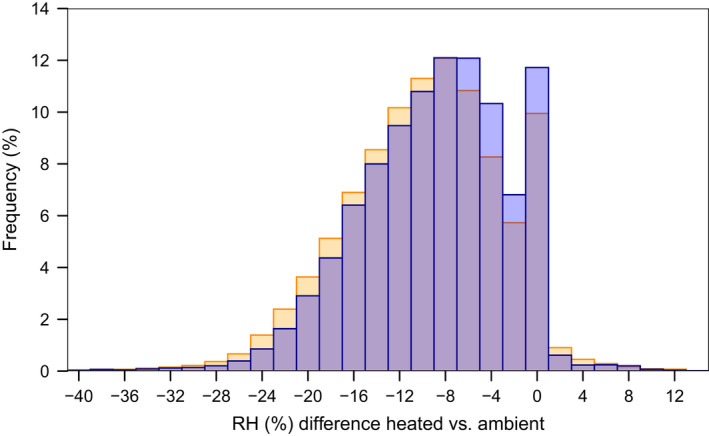
Frequency of the measured difference in relative humidity between the 10 climate‐warming chambers (set temperature increase: 3 K) and ambient rainforest conditions during the day (6 a.m.–6 p.m.), in yellow, and night, in blue. Purple bars indicate an overlap. Frequencies were calculated based on measurements taken every 15 min for 456 days (18‐05‐2017 to 17‐08‐2018)

**FIGURE 7:**
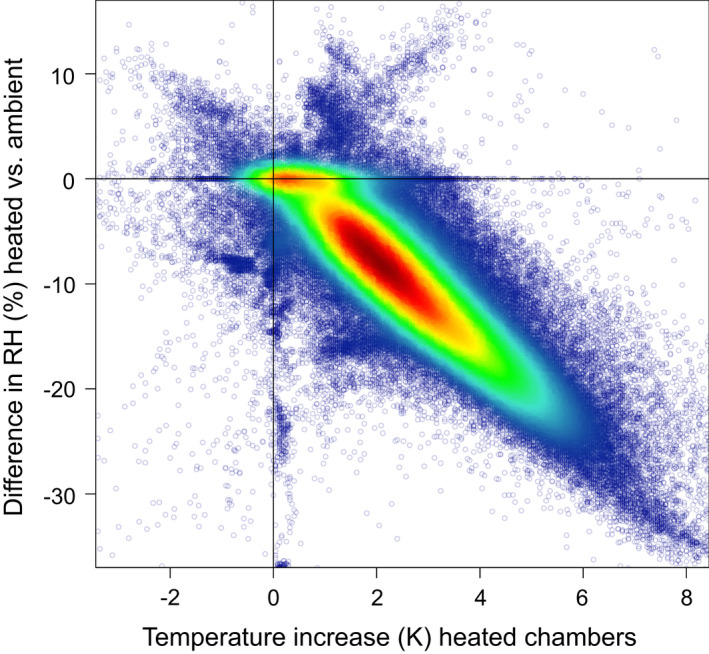
Change in relative humidity compared to ambient conditions (ΔRH, in % humidity) in the 10 heated chambers relative to the achieved warming (ΔTemp, in K). Points are color coded by point density, with very high densities shown in red. Heated chambers have higher temperatures and lower RH. Spearman correlation between ΔRH and ΔTemp: rho = −0.86. Shown are data from all heated chambers combined (heating and heating + CO_2_ addition), measured at 15‐min intervals for 456 days (18‐05‐2017 to 17‐08‐2018)

**FIGURE A12:**
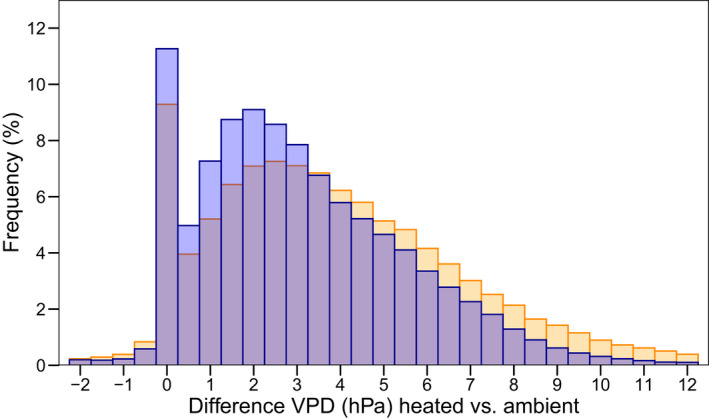
Histogram of difference in vapor pressure deficit (VPD), in 0.5‐hPa increments, between 10 heated chambers and ambient conditions in a warming experiment using open‐top chambers in the rainforest understorey at La Selva, Costa Rica. Positive values indicate higher VPD in the heated chambers than outside, i.e. drier conditions inside the chambers. Frequencies were calculated based on measurements taken every 15 min for 456 days (18‐05‐2017 to 17‐08‐2018)

**FIGURE A13:**
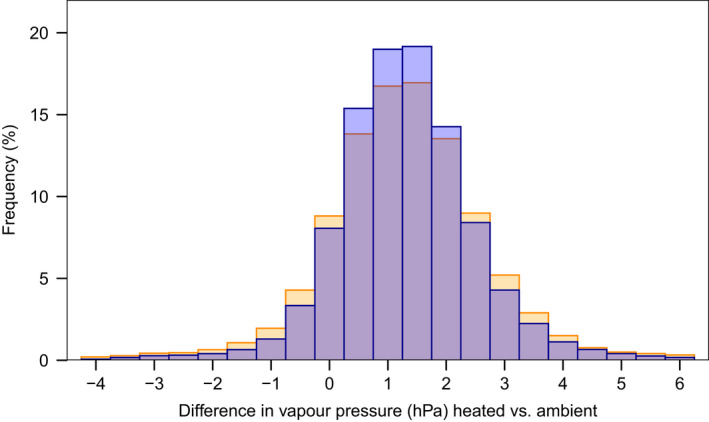
Histogram of difference in vapor pressure (Pw), in 0.5‐hPa increments, between 10 heated chambers and ambient conditions in a warming experiment using open‐top chambers in the rainforest understorey at La Selva, Costa Rica. Positive values indicate higher Pw in the heated chambers than outside. Frequencies were calculated based on measurements taken every 15 min for 456 days (18‐05‐2017 to 17‐08‐2018)

**FIGURE A14:**
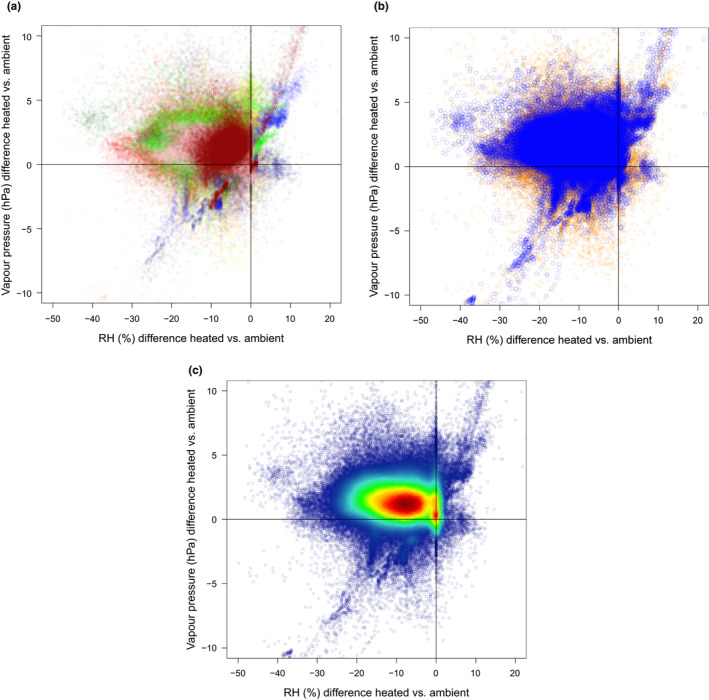
FIG Difference in vapor pressure (Pw, in hPa) versus difference in relative humidity (in %) of 10 heated chambers compared to ambient conditions in a warming experiment using open‐top chambers in the rainforest understorey at La Selva, Costa Rica. Points colored by chamber. Points are colored by chamber (a), day and night (Orange and blue, respectively, b), or point density (heat map, c). In (c) the colours indicate the point density, with very high densities shown in red, representing a decrease in RH and a concurrent increase in Pw, i.e. a lower relative but higher absolute humidity. Shown are data from all heated chambers combined (heating and heating + CO_2_ addition), measured at 15 min intervals for 456 days (18‐05‐2017 to 17‐08‐2018)

**FIGURE A15:**
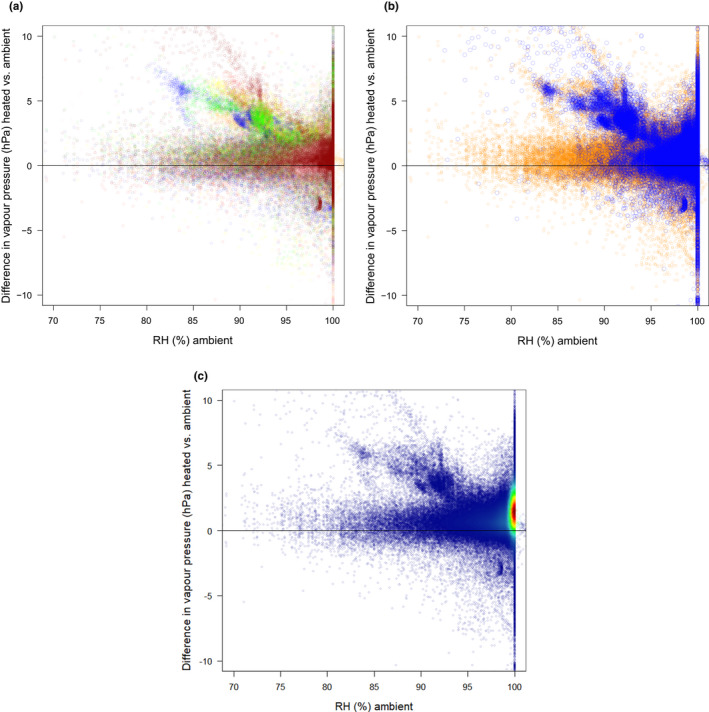
Difference in vapor pressure (Pw, in hPa) of 10 heated chambers compared to ambient conditions (*y* axis) versus ambient relative humidity (in %, *x* axis) in a warming experiment using open‐top chambers in the rainforest understorey at La Selva, Costa Rica. Points are colored by chamber (a), day and night (Orange and blue, respectively, b), or point density (heat map, c). In c points are colour‐coded by point density, with very high densities shown in red, occurring at RH 100% with mostly positive deviations of Pw, i.e. heated chambers had higher absolute humidity. Shown are data from all heated chambers combined (heating and heating + CO_2_ addition), measured at 15 min intervals for 456 days (18‐05‐2017 to 17‐08‐2018)

**FIGURE A16:**
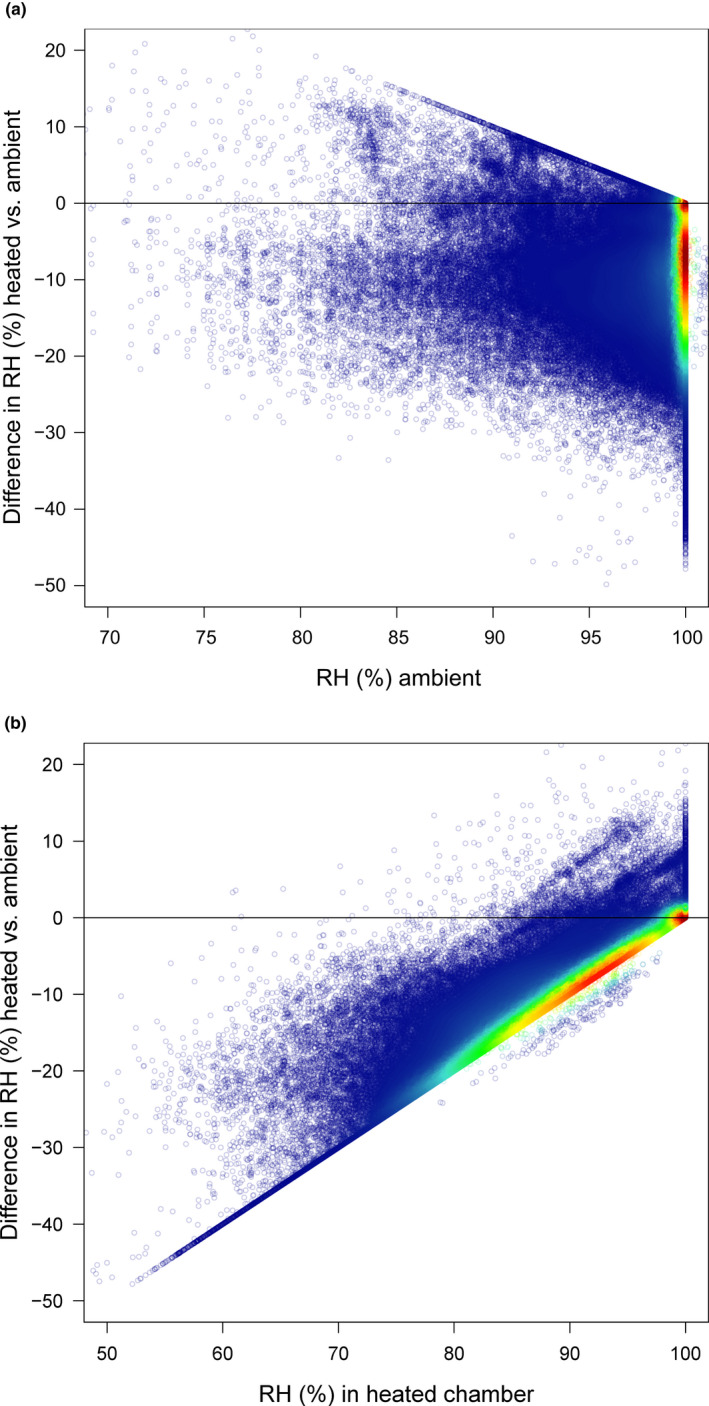
Difference in relative humidity (RH, in %) of 10 heated chambers compared to ambient conditions (*y* axis) versus relative humidity (in %, *x* axis) in ambient air (b) or inside the heated chambers (b) in a warming experiment using open‐top chambers in the rainforest understorey at La Selva, Costa Rica. Points are colour‐coded by point density, with very high densities shown in red, occurring at ambient RH near 100%, with reductions in RH mostly at 0% (with RH at 100% inside and outside of the chambers) or between 3 and 17%, i.e. heated chambers had lower relative humidity. Shown are data from all heated chambers combined (heating and heating + CO_2_ addition), measured at 15 min intervals for 456 days (18‐05‐2017 to 17‐08‐2018)

The absolute humidity (vapor pressure) was most frequently higher by about 1.5 hPa (mostly between 0 and +2.5 hPa) in the heated chambers than outside (Figures A13, A14, A15). However, the heated chambers were still dryer because due to the higher temperatures and consequently higher saturation vapor pressure, RH decreased and the vapor pressure deficit (VPD) increased (Figure 6, Figure A12). RH decreased on average by between 6% and 15% (and VPD increased by between 2 and 6 hPa) in the different chambers, with stronger decreases in chambers with more heating (Spearman´s rho = −.86, Figure 7, Figure A9, Table 1). This RH decrease was not correlated with the ambient RH (rho = .06, Figures A9 and A16). The difference was larger during the day than during the night (Figure 5, Figures A14b and A15b).

The reduction in RH was most frequent either around 0 or above 4%, with a dip in between these values (Figure 6). This phenomenon was of course reflected in the VPD pattern (Figure A12), but was not observed for the absolute humidity (Figures A13, A14, A15). Rather than being an artefact, this phenomenon reflects a bimodal distribution in VPD and RH inside the chambers, with a peak around 100% (caused by evapotranspiration inside the chambers, raising humidity close to the dew point), and a lower range when humidity was more dependent on the humidity outside (Figure 7, Figures A14, A15, A16). In the heated chambers, RH would frequently not approach 100% during the night (Figure 4, Figure A7b), which strongly contrasts with the ambient situation (although even outside one of the sensors registered values below 100% RH at night (Figure A7b), which again might indicate that part of the variability was due to sensor inaccuracies).

### 3.4 CO_2_ levels

In the chambers subjected to CO_2_ fertilization, the mean CO_2_ level was considerably and significantly higher than that of the outside air, by between 131 and 402 ppm on average, depending on the chamber (Table 2), with an average between chambers of 250 ppm. Interestingly, the CO_2_ increase was higher in the non‐heated than in the heated chambers (ANOVA, *F*(1, 8) = 5.51, *p* < .05). This can probably be explained by the accelerated air renewal in the heated chambers due to the warm air flowing out of the chamber faster than the air at ambient temperature. This effect could not be totally countered by the higher CO_2_ addition in the heated chambers. Variation between individual chambers could have been caused by differences in the output pressure of the CO_2_ pressure regulators or different resistances in the paths from the gas bottles to the chambers, and by the chambers trapping CO_2_ naturally produced by respiration processes.

## 4 DISCUSSION

We tested a set‐up with open‐top chambers to study the separate and combined effects of warming and increased CO_2_ concentrations on plants under field conditions in a tropical rainforest. The chambers themselves had a minimal effect on microclimatic conditions while the heating and CO_2_ treatments were very effective. The main undesired effect was a decrease in relative humidity associated with warming. This decrease is due to basic biophysical laws and cannot be avoided unless moisture is added artificially, which would greatly increase the complexity of the experimental setup. Whether the effects on the experimental outcomes are a problem needs to be evaluated for each research question individually. What is clear is that it does not fully reflect the effect of global warming on air humidity in the rainforest understorey. Even though the air over the continents might become drier under global warming (Sherwood & Fu, 2014), near‐surface relative humidity is expected to remain more or less the same (Li et al., 2018). At least in the rainforest understorey, local evapotranspiration should maintain the typically low VPDs in this environment, but only as long as precipitation remains sufficient to create a permanently humid soil (Wright, 1991).

Our experiment was as successful in achieving the target temperature increase as the active “forced air” warming experiments reviewed in Ettinger et al. (2019), which achieved between 49% and 95% of the targeted temperature increase, as averages of various studies. Over time, our individual chambers reached, on average, between 60% and 127% of the targeted 3 K, with an overall average 80%. Seeing the variability between chambers, which is common also in other warming experiments, a regression‐type analysis of warming results on biological processes based on the actual measured temperature differences, rather than an ANOVA‐type analysis based on the target values, may improve the detection of warming effects (Ettinger et al., 2019). Taking this one step further, the experiment could be designed to represent a gradient of warming levels (Pelini et al., 2011).

More important than the overall mean temperature increase is its temporal stability. In our experiment, most chambers suffered failures of the temperature regulation at some point during the experiment and we also experienced several electricity cuts. Unsurprisingly, the temperature difference disappeared during these events, but while in running mode the system produced reliable and stable temperature differences. For future setups, we advise installing an emergency generator to bridge periods with power cuts. For the CO_2_ concentration, our measurements were not continuous, but our weekly samples also showed a stable increase, in spite of the simplicity of the system without a feedback regulation for the CO_2_ input. This stability was probably aided by the sheltered conditions in our study site, typical for lowland rainforest understoreys (Lakatos et al., 2012; Leigh, 1999), providing a relatively constant low level of turbulence and thus a constant renewal rate for the air from the chambers.

One previous experiment has provided rainforest seedlings with increased CO_2_ (Würth et al., 1998a), although not with simultaneous warming. Our setup differs from theirs in the open tops of the chambers (instead of tents). Open tops caused a greater loss of CO_2_ but had the great advantage that rain could enter the chambers, so that they did not need to be watered manually. This did not only save effort but also assured a natural spatial distribution of the rainwater, which was especially important as our study objects were poikilohydric epiphytes.

Comparing our system to the only other rainforest warming system in operation (although without CO_2_ enrichment) based on infrared heating (Kimball et al., 2018), we can evaluate the pros and cons of both systems. In our system, heating was achieved via the air and the heating of surfaces was indirect via convective warming. In their system, surfaces were heated directly and the heating of the air (not registered) was indirect via convective warming from these surfaces. Both systems cause an additional drying, a common phenomenon in warming systems that do not actively add humidity (Ettinger et al., 2019). We registered changes in humidity for air and artificial leaves but not for soils, while they did only for soils. We can thus not compare the severity of these effects directly, but it is likely that the decrease in air humidity was the same for every K of air heating in both systems. Their system will have had less heating of the air overall, but air closer to the leaves would be warmed and thus be drier. On the other hand, our soils will have been warmed less (not registered) and thus will have dried out less than with the surface warming caused by infrared heaters. In our case, as our focal organisms were bryophytes, for whom daily hydration patterns as well as moisture gradients within the moss canopy affect their metabolic activity, the heterogeneous drying patterns caused by surface heating would be unacceptable. However, for other applications, this may be less critical.

In our system, an addition of moisture would be possible by adding it into the air moving into the chamber, as used in some more elaborate chamber systems (Tingey et al., 1996), while for an infrared heating system, there would be no natural way of adding humidity. Irrigation has been suggested as a method of offsetting the drop in air humidity or faster soil drying in infrared systems (Kimball, 2011). If the distribution of water between soil and air is not relevant for the processes studied, this approach may be appropriate, but irrigating the soil would hardly help epiphytic plants. The alternative, a direct supply of water to the epiphytes, would add a strong experimental factor that would be hard to relate directly to climate warming. For this reason, we did not water our bryophytes to compensate for the lower air humidity. Adding an air humidifier in the system would have been expensive and complicated, but with our experience with this experiment we would now strongly recommend investing in such an improvement.

An advantage of the infrared warming system used by Kimball et al. (2018) is the absence of chambers, which allows wind and animals to move freely through the experimental setup (if not scared off by the installations). This is an advantage if, for example, herbivory or seedling establishment from naturally dispersed seeds is among the studied processes. In general, their system is easier to scale up to larger areas, being thus more suitable for ecosystem‐level studies. However, for mimicking climate change realistically, we consider that in the radiation‐poor environment of a forest understorey, radiative heating is less appropriate than in exposed ecosystems, while the disadvantages of chambers are less pronounced. If, in addition to warming, CO_2_ enrichment is one of the treatments of interest, the advantage of chambers becomes even larger. Although free‐air alternatives are possible for both warming and CO_2_ enrichment, that option would be a lot more technical and expensive to install and maintain for small plots like ours. However, these open‐top chambers cannot be scaled up to entire forests, where a very large chamber would be needed that would need to have a (partial) roof to effectively contain both the heat and CO_2_. Therefore, the choice of a warming and CO_2_ fertilization method depends not only on the processes studied but also strongly on the scale of interest. Our chambers are appropriate in small plots (up to a few m in diameter) and infrared heating with FACE in larger plots.

## 5 CONCLUSIONS

Actively heating and CO_2_‐fertilizing tropical understorey epiphytes or tree seedlings by leading warmed air and CO_2_ into open‐top chambers is a viable method to study climate‐change effects on plants in tropical rainforest understoreys. Control chambers had a minimal effect on microclimatic conditions, but with heating the relative humidity dropped. Since under natural climate warming understorey air humidity will probably remain high, we recommend adding a humidifier to the warmed air stream in future set ups to avoid changes in humidity as a confounding factor. For larger‐scales studies, which address ecosystem‐ rather than plant‐level processes, infrared heating combined with free‐air CO_2_ enrichment may provide a good alternative. However, in the rainforest understorey, which naturally has very low radiation levels, heated chambers offer clear advantages over infrared heating, providing a higher realism while hardly suffering from microclimatic chamber effects.

## CONFLICT OF INTEREST

None declared.

## AUTHOR CONTRIBUTIONS

**Maaike Y. Bader:** Conceptualization (equal); Formal analysis (supporting); Funding acquisition (equal); Methodology (equal); Project administration (equal); Supervision (equal); Visualization (supporting); Writing – original draft (lead); Writing – review & editing (equal). **Elodie Moureau:** Data curation (lead); Formal analysis (equal); Investigation (equal); Project administration (supporting); Visualization (equal); Writing – original draft (supporting); Writing – review & editing (supporting). **Nada Nikolić:** Data curation (supporting); Formal analysis (equal); Investigation (equal); Validation (lead); Visualization (lead); Writing – review & editing (supporting). **Thomas Madena:** Methodology (equal); Validation (supporting); Writing – review & editing (supporting). **Nils Koehn:** Methodology (equal). **Gerhard Zotz:** Conceptualization (equal); Funding acquisition (equal); Methodology (equal); Project administration (equal); Supervision (equal); Writing – review & editing (equal).

## Supporting information

Appendix S1

## Data Availability

Technical data are available in the Appendix of this paper. Gerber files for the electronic circuits and details of the materials used (types, suppliers, costs) are provided as Supporting Information. Microclimatic data are available on Dryad at https://doi.org/10.5061/dryad.qv9s4mwfv.

## APPENDIX A

**TABLE A1a:** Hourly means (hour shown + 1 h) during a dry season month (May 2018) of the temperature inside and outside of five control chambers in a warming experiment using open‐top chambers in the rainforest understorey at La Selva, Costa Rica. Shown are means, standard deviations (between chambers, calculated per day and shown as the mean of all days; not shown for outside measurements due to the low replication of sensors), and the number of comparisons (in this month we had two outside sensors, used in five pairwise comparisons, each chamber paired with the nearest reference point) and results of paired *t* tests comparing the hourly means inside and outside the chambers (no significant differences were found). See Figure 3a in the paper for a graphical representation of the results

Hour	Mean T in (°C)	*SD*	Mean T out (°C)	*n*	*t*	*p*
00	23.4	0.1	23.4	5	0.632	.562
01	23.2	0.1	23.2	5	0.618	.570
02	23.1	0.1	23	5	0.354	.741
03	22.9	0.1	22.9	5	0.659	.546
04	22.7	0.1	22.7	5	0.261	.807
05	22.6	0.1	22.6	5	0.995	.376
06	22.9	0.1	22.8	5	1.497	.209
07	23.6	0.1	23.5	5	1.265	.274
08	24.8	0.3	24.8	5	0.447	.678
09	25.8	0.4	26	5	−1.309	.261
10	26.5	0.4	27	5	−2.217	.091
11	27.1	0.5	27.5	5	−1.621	.180
12	27.5	0.7	27.9	5	−1.018	.366
13	27.6	0.6	27.9	5	−1.263	.275
14	27.3	0.4	27.4	5	−0.593	.585
15	26.8	0.2	26.8	5	−0.711	.516
16	26.2	0.2	26.3	5	−0.345	.748
17	25.6	0.2	25.5	5	0.416	.699
18	25.0	0.3	24.9	5	0.957	.393
19	24.6	0.3	24.5	5	1.097	.334
20	24.3	0.3	24.2	5	1.123	.324
21	24.1	0.3	24.0	5	1.078	.342
22	23.9	0.2	23.8	5	0.902	.418
23	23.7	0.1	23.6	5	0.987	.380

**TABLE A1b:** Hourly means (hour shown + 1 h) during a wet season month (July 2018) of the temperature inside and outside of five control chambers in a warming experiment using open‐top chambers in the rainforest understorey at La Selva, Costa Rica. Shown are means, standard deviations (between chambers, calculated per day and shown as the mean of all days; not shown for outside measurements due to the low replication of sensors), and the number of comparisons (in this month we had two outside sensors, used in five pairwise comparisons, each chamber paired with the nearest reference point) and results of paired *t* tests comparing the hourly means inside and outside the chambers (significant differences (*p* < .05) are highlighted in bold). See Figure 3b in the paper for a graphical representation of the results

Hour	Mean T in (°C)	*SD*	Mean T out (°C)	*t*	*p*
00	23.5	0.2	23.3	2.780	.050
01	23.4	0.2	23.2	2.624	.059
02	23.3	0.2	23.2	2.589	.061
03	23.2	0.2	23.0	2.911	.**044**
04	23.2	0.2	23.0	2.960	.**042**
05	23.1	0.2	22.9	2.902	.**044**
06	23.2	0.2	23.0	3.007	.**040**
07	23.5	0.2	23.3	2.475	.069
08	23.9	0.2	23.8	1.208	.293
09	24.4	0.3	24.3	0.408	.704
10	24.7	0.3	24.8	−0.200	.851
11	25.1	0.4	25.1	−0.390	.717
12	25.2	0.4	25.3	−0.383	.721
13	25.3	0.4	25.3	−0.243	.820
14	25.2	0.3	25.2	0.114	.915
15	25.1	0.2	25.0	0.948	.397
16	24.9	0.2	24.8	1.299	.264
17	24.8	0.2	24.6	1.632	.178
18	24.5	0.2	24.3	2.126	.101
19	24.2	0.2	24.0	2.539	.064
20	24.0	0.2	23.8	2.658	.056
21	23.9	0.2	23.7	2.738	.052
22	23.8	0.2	23.6	2.678	.055
23	23.7	0.2	23.5	2.710	.054

**TABLE A2a:** Hourly means (hour shown + 1 h) during a dry season month (May 2018) of the relative humidity inside and outside of five control chambers in a warming experiment using open‐top chambers in the rainforest understorey at La Selva, Costa Rica. Shown are means, standard deviations (between chambers, calculated per day and shown as the mean of all days; not shown for outside measurements due to the low replication of sensors), and the number of comparisons (in this month we had two outside sensors, used in five pairwise comparisons, each chamber paired with the nearest reference point) and results of paired *t* tests comparing the hourly means inside and outside the chambers (significant differences (*p* < .05) are highlighted in bold). See Figure 4a in the paper for a graphical representation of the results

Hour	Mean RH in (%)	*SD*	Mean RH out (%)	*n*	*t*	*p*
00	99.1	1.5	99.7	5	−0.949	.396
01	99.2	1.4	99.7	5	−1.016	.367
02	99.2	1.4	99.7	5	−1.036	.359
03	99.2	1.4	99.8	5	−1.079	.341
04	99.2	1.4	99.8	5	−1.051	.352
05	99.2	1.4	99.8	5	−1.069	.345
06	99.2	1.4	99.9	5	−1.203	.295
07	99.2	1.3	99.9	5	−1.222	.289
08	99.0	1.4	99.7	5	−1.231	.286
09	98.1	1.5	98.0	5	0.158	.882
10	96.8	1.8	95.6	5	1.613	.182
11	95.7	1.9	93.6	5	2.781	.050
12	94.9	2.0	92.4	5	2.800	.**049**
13	94.9	1.7	92.5	5	3.358	.**028**
14	95.7	1.3	94.2	5	3.264	.**031**
15	96.9	1.4	96.2	5	1.575	.190
16	97.5	1.6	97.2	5	0.618	.570
17	98.0	1.9	97.9	5	0.063	.953
18	98.4	2.0	98.4	5	−0.109	.919
19	98.6	2.0	98.8	5	−0.285	.790
20	98.8	1.9	99.1	5	−0.502	.642
21	99.0	1.7	99.4	5	−0.693	.526
22	99.1	1.5	99.5	5	−0.778	.480
23	99.1	1.5	99.6	5	−0.886	.426

**TABLE A2b:** Hourly means (hour shown + 1 h) during a wet season month (July 2018) of the relative humidity inside and outside of five control chambers in a warming experiment using open‐top chambers in the rainforest understorey at La Selva, Costa Rica. Shown are means, standard deviations (between chambers, calculated per day and shown as the mean of all days; not shown for outside measurements due to the low replication of sensors), and the number of comparisons (in this month we had two outside sensors, used in five pairwise comparisons, each chamber paired with the nearest reference point) and results of paired *t* tests comparing the hourly means inside and outside the chambers (no significant differences were found). See Figure 4b in the paper for a graphical representation of the results

Hour	Mean RH in (%)	*SD*	Mean RH out (%)	*n*	*t*	*p*
00	99.7	0.6	100	5	−0.8	.469
01	99.7	0.6	100	5	−0.783	.477
02	99.8	0.6	100	5	−0.792	.473
03	99.8	0.6	100	5	−0.796	.471
04	99.8	0.6	100	5	−0.783	.477
05	99.8	0.5	100	5	−0.781	.478
06	99.8	0.5	100	5	−0.772	.483
07	99.8	0.5	100	5	−0.753	.493
08	99.8	0.5	100	5	−0.797	.470
09	99.8	0.5	100	5	−0.766	.486
10	99.8	0.5	99.9	5	−0.491	.649
11	99.7	0.5	99.8	5	−0.33	.758
12	99.8	0.5	99.7	5	0.395	.713
13	99.7	0.5	99.6	5	0.616	.572
14	99.7	0.5	99.6	5	0.464	.667
15	99.7	0.5	99.7	5	0.164	.878
16	99.7	0.6	99.8	5	−0.373	.728
17	99.7	0.6	99.9	5	−0.606	.577
18	99.6	0.8	99.9	5	−0.775	.482
19	99.6	0.9	100	5	−0.856	.440
20	99.6	1	100	5	−0.89	.424
21	99.7	0.6	100	5	−0.825	.456
22	99.7	0.6	100	5	−0.823	.457
23	99.7	0.6	100	5	−0.817	.460

**TABLE A2c:** Hourly means (hour shown + 1 h) during April 2018 of relative humidity (RH) inside outside of five control chambers in a warming experiment using open‐top chambers in the rainforest understorey at La Selva, Costa Rica. Shown are means, standard deviations (between chambers, calculated per day and shown as the mean of all days; not shown for outside measurements due to the low replication of sensors), and the number of comparisons (in this month we had two outside sensors, used in five pairwise comparisons, each chamber paired with the nearest reference point) and results of paired *t*‐tests (significant differences (*p* < .05) are highlighted in bold). See Figure A7a for a graphical representation of the results

Hour	Mean RH in (%)	*SD*	Mean RH out (%)	*n*	*t*	*p*
00	99.7	0.6	99.7	5	0.162	.879
01	99.7	0.6	99.7	5	0.094	.930
02	99.7	0.6	99.7	5	0.057	.958
03	99.7	0.6	99.8	5	−0.093	.930
04	99.7	0.6	99.8	5	−0.134	.900
05	99.8	0.5	99.8	5	−0.088	.934
06	99.8	0.5	99.8	5	−0.282	.792
07	99.7	0.6	99.7	5	−0.022	.983
08	99.4	0.9	99.4	5	−0.005	.996
09	98.0	1.5	97.7	5	0.556	.608
10	96.4	1.7	95.4	5	1.745	.156
11	94.6	1.6	93.2	5	3.387	.**028**
12	93.7	1.7	92.2	5	3.042	.**038**
13	94.0	1.2	92.6	5	3.080	.**037**
14	95.0	0.7	93.9	5	3.087	.**037**
15	96.5	0.6	95.4	5	2.748	.052
16	97.6	0.8	96.9	5	1.984	.118
17	98.2	0.9	97.4	5	2.046	.110
18	98.6	0.9	98.0	5	1.711	.162
19	99.0	0.9	98.6	5	1.306	.262
20	99.2	0.9	98.9	5	0.911	.414
21	99.5	0.8	99.2	5	0.706	.519
22	99.6	0.7	99.5	5	0.410	.703
23	99.7	0.7	99.6	5	0.206	.847

**TABLE A3:** Hourly means (hour shown + 1 h) of leaf wetness inside five control chamber and five outside plots in a warming experiment using open‐top chambers in the rainforest understorey at La Selva, Costa Rica. Shown are means, with standard deviations, and the number of comparisons and results of unpaired *t*‐tests (significant differences (*p* < .05) are highlighted in bold). The units are the % of leaf that is wet. As this is 0% or 100% most of the time, the mean indicates the wetness duration. Data from May 2017, before the artificial leaves of the sensors become overgrown with algae. See Figure A8 for a graphical representation of the results

Hour	Mean leaf‐wetness in (%)	*SD*	Mean leaf‐wetness out (%)	*SD*	*n*	*t*	*p*
00	88.1	5.9	76.2	11.8	5	2.013	.092
01	88.6	5.6	76.6	11.8	5	2.050	.088
02	89.2	5.3	78.0	11.8	5	1.935	.105
03	88.9	5.1	77.8	10.9	5	2.042	.090
04	87.9	5.2	76.4	10.4	5	2.208	.070
05	87.8	4.9	76.3	10.5	5	2.231	.070
06	86.9	4.7	75.4	9.2	5	2.484	.**048**
07	78.7	5.5	67.2	6.0	5	3.174	.**013**
08	59.8	10.2	51.0	7.7	5	1.536	.166
09	45.7	11.1	39.3	9.8	5	0.965	.363
10	40.5	10.4	36.0	7.2	5	0.801	.449
11	34.3	8.1	30.5	7.0	5	0.795	.450
12	35.8	9.1	30.6	5.7	5	1.075	.319
13	33.7	9.1	28.5	6.3	5	1.057	.325
14	45.9	10.0	37.1	6.2	5	1.674	.140
15	54.6	8.6	44.2	5.7	5	2.246	.060
16	65.1	9.4	53.0	5.9	5	2.432	.**047**
17	72.9	8.2	59.0	7.2	5	2.842	.**022**
18	76.4	7.8	61.1	8.7	5	2.929	.**019**
19	80.2	7.3	65.2	9.0	5	2.888	.**021**
20	84.7	6.6	69.9	9.8	5	2.817	.**026**
21	86.6	6.6	72.3	10.4	5	2.604	.**036**
22	88.2	5.8	74.7	11.2	5	2.398	.053
23	89.3	5.4	75.9	11.9	5	2.294	.065

**TABLE A4a:** Hourly means (hour shown + 1 h) of photosynthetically‐active radiation (PAR, in µmol m^−2^ s^−1^) inside three open‐top chambers (PAR in) and in three outside plots (PAR out) in a tropical rain forest in Costa Rica, with standard deviations and the number of comparisons and results of unpaired *t*‐tests for the mean hourly values based on the whole period of measurements (187–244 days, depending on the sensor). We tested only the hours in which there is light. No significant differences were found, but see (b–d) for tests of differences for single inside‐outside pairs

Hour	Mean PAR in	*SD*	Mean PAR out	*SD*	*n*	*t*	*p*
06	0.2	0.2	0.4	0.3	3	−0.761	.494
07	2.8	0.4	2.9	1.1	3	−0.187	.865
08	11.7	8.5	9.8	1.2	3	0.369	.746
09	18.5	13.1	26.7	25.0	3	−0.503	.649
10	16.7	11.8	16.7	3.7	3	0.000	1.000
11	13.6	5.5	19.7	9.5	3	−0.960	.404
12	14.1	8.4	16.4	7.3	3	−0.356	.740
13	12.8	1.8	15.9	8.8	3	−0.600	.605
14	6.0	2.2	10.8	2.7	3	−2.352	.081
15	3.2	1.4	4.8	1.4	3	−1.421	.228
16	0.6	0.4	1.0	0.3	3	−1.469	.223
17	0.0	0.0	0.0	0.0	3	−0.953	.424

**TABLE A4b:** Hourly means of PAR inside and outside of chamber Con‐4, with days as replicates for the paired *t*‐tests. Significant results (*p* < .05) are highlighted in bold. PAR is sometimes higher inside and sometimes outside the chamber

Hour	Mean PAR in	*SD*	Mean PAR out	*SD*	*n*	*t*	*p*
06	0.3	0.5	0.1	0.2	187	5.760	**<.001**
07	2.5	2.2	1.7	1.5	187	7.151	**<.001**
08	11.9	18.4	9.7	26.3	187	0.908	.365
09	14.3	15.8	10.8	15.1	187	2.265	.**025**
10	18.5	31.2	17.0	34.5	187	0.548	.584
11	11.7	8.6	12.1	16.0	187	−0.343	.732
12	13.3	21.0	10.3	7.0	185	1.853	.066
13	10.9	14.5	10.0	8.7	185	0.808	.420
14	6.1	4.2	7.6	12.2	186	−2.024	.**044**
15	4.0	7.4	3.5	4.0	186	1.111	.268
16	0.8	1.1	0.7	1.0	187	1.847	.066
17	0.0	0.2	0.0	0.0	187	1.675	.096

**TABLE A4c:** Hourly means of PAR inside and outside of chamber C4, with days as replicates for the paired *t*‐tests. Significant results (*p* < .05) are highlighted in bold. Only for this chamber is PAR consistently higher outside than inside the chamber (compare Tables A4b, A4c and d)

Hour	Mean PAR in	*SD*	Mean PAR out	*SD*	*n*	*t*	*p*
06	0.0	0.1	0.6	1.0	244	−9.439	**<.001**
07	2.6	10.7	3.8	3.9	244	−1.650	.100
08	3.0	5.2	8.7	7.4	244	−11.010	**<.001**
09	8.1	14.7	55.5	113.8	244	−6.921	**<.001**
10	4.2	2.4	12.9	9.0	244	−18.617	**<.001**
11	9.2	21.7	16.4	12.9	244	−5.046	**<.001**
12	6.2	6.0	14.4	11.6	243	−12.485	**<.001**
13	13.1	31.7	26.0	49.3	243	−3.533	**<.001**
14	3.8	8.0	12.2	15.6	244	−7.940	**<.001**
15	1.6	3.2	6.3	11.3	244	−7.275	**<.001**
16	0.1	0.3	1.3	1.7	244	−11.811	**<.001**
17	0.0	0.0	0.1	0.3	244	−4.221	**<.001**

**TABLE A4d:** Hourly means of PAR inside and outside of chamber Con‐3, with days as replicates for the paired *t*‐tests. Significant results (*p* < .05) are highlighted in bold. PAR is sometimes higher inside and sometimes outside the chamber

Hour	Mean PAR in	*SD*	Mean PAR out	*SD*	*n*	*t*	*p*
06	0.4	0.7	0.5	1.1	244	−1.257	.209
07	3.3	2.3	3.3	2.6	244	0.044	.965
08	20.1	39.9	11.1	19.5	244	4.29	**<.001**
09	33.2	79.4	13.8	15.2	244	5.188	**<.001**
10	27.5	54.1	20.3	29.3	244	2.406	.017
11	19.7	23.8	30.4	57.0	244	−3.659	**<.001**
12	22.9	45.8	24.6	50.0	244	−0.731	.465
13	14.4	23.7	11.8	14.7	244	2.046	.041
14	8.2	9.4	12.6	17.7	244	−5.242	**<.001**
15	3.8	4.3	4.6	7.1	244	−2.232	.026
16	0.8	1.4	1.0	1.4	244	−4.656	**<.001**
17	0.0	0.0	0.0	0.1	244	−2.546	.011
